# Local variation in childhood diarrheal morbidity and mortality in Africa, 2000-2015

**DOI:** 10.1056/NEJMoa1716766

**Published:** 2018-09-20

**Authors:** Robert C. Reiner, Nicholas Graetz, Daniel C. Casey, Christopher Troeger, Gregory M. Garcia, Jonathan F. Mosser, Aniruddha Deshpande, Scott J. Swartz, Scott J. Swartz, Sarah E. Ray, Brigette F. Blacker, Puja C. Rao, Aaron Osgood -Zimmerman, Roy Burstein, David M. Pigott, Ian M. Davis, Ian D. Letourneau, Lucas Earl, Jennifer M. Ross, Ibrahim A. Khalil, Tamer H. Farag, Oliver J. Brady, Moritz U.G. Kraemer, David L. Smith, Samir Bhatt, Daniel J. Weiss, Peter W. Gething, Nicholas J. Kassebaum, Ali H. Mokdad, Christopher J.L. Murray, Simon I. Hay

**Affiliations:** Institute for Health Metrics and Evaluation, University of Washington, 2301 5th Ave Suite 600, Seattle, WA 98121, USA; Institute for Health Metrics and Evaluation, University of Washington, 2301 5th Ave Suite 600, Seattle, WA 98121, USA; Institute for Health Metrics and Evaluation, University of Washington, 2301 5th Ave Suite 600, Seattle, WA 98121, USA; Institute for Health Metrics and Evaluation, University of Washington, 2301 5th Ave Suite 600, Seattle, WA 98121, USA; Institute for Health Metrics and Evaluation, University of Washington, 2301 5th Ave Suite 600, Seattle, WA 98121, USA; Institute for Health Metrics and Evaluation, University of Washington, 2301 5th Ave Suite 600, Seattle, WA 98121, USA; Division of Pediatric Infectious Diseases, Seattle Children’s Hospital, Seattle, WA, USA; Institute for Health Metrics and Evaluation, University of Washington, 2301 5th Ave Suite 600, Seattle, WA 98121, USA; Institute for Health Metrics and Evaluation, University of Washington, 2301 5th Ave Suite 600, Seattle, WA 98121, USA; Institute for Health Metrics and Evaluation, University of Washington, 2301 5th Ave Suite 600, Seattle, WA 98121, USA; Institute for Health Metrics and Evaluation, University of Washington, 2301 5th Ave Suite 600, Seattle, WA 98121, USA; Institute for Health Metrics and Evaluation, University of Washington, 2301 5th Ave Suite 600, Seattle, WA 98121, USA; Institute for Health Metrics and Evaluation, University of Washington, 2301 5th Ave Suite 600, Seattle, WA 98121, USA; Institute for Health Metrics and Evaluation, University of Washington, 2301 5th Ave Suite 600, Seattle, WA 98121, USA; Institute for Health Metrics and Evaluation, University of Washington, 2301 5th Ave Suite 600, Seattle, WA 98121, USA; Institute for Health Metrics and Evaluation, University of Washington, 2301 5th Ave Suite 600, Seattle, WA 98121, USA; Institute for Health Metrics and Evaluation, University of Washington, 2301 5th Ave Suite 600, Seattle, WA 98121, USA; Institute for Health Metrics and Evaluation, University of Washington, 2301 5th Ave Suite 600, Seattle, WA 98121, USA; Institute for Health Metrics and Evaluation, University of Washington, 2301 5th Ave Suite 600, Seattle, WA 98121, USA; Institute for Health Metrics and Evaluation, University of Washington, 2301 5th Ave Suite 600, Seattle, WA 98121, USA; Division of Allergy and Infectious Diseases, Department of Medicine, University of Washington, Seattle, WA, USA; Institute for Health Metrics and Evaluation, University of Washington, 2301 5th Ave Suite 600, Seattle, WA 98121, USA; Institute for Health Metrics and Evaluation, University of Washington, 2301 5th Ave Suite 600, Seattle, WA 98121, USA; Department of Infectious Disease Epidemiology, London School of Hygiene & Tropical Medicine, London, UK; Computational Epidemiology Lab, Boston Children’s Hospital, Boston, MA, USA; Harvard Medical School, Harvard University, Boston, MA, USA; Department of Zoology, University of Oxford, Oxford, UK; Institute for Health Metrics and Evaluation, University of Washington, 2301 5th Ave Suite 600, Seattle, WA 98121, USA; Big Data Institute, Li Ka Shing Centre for Health Information and Discovery, University of Oxford, Oxford, UK; Department of Infectious Disease Epidemiology, Imperial College London, London, UK; Big Data Institute, Li Ka Shing Centre for Health Information and Discovery, University of Oxford, Oxford, UK; Department of Infectious Disease Epidemiology, Imperial College London, London, UK; Big Data Institute, Li Ka Shing Centre for Health Information and Discovery, University of Oxford, Oxford, UK; Institute for Health Metrics and Evaluation, University of Washington, 2301 5th Ave Suite 600, Seattle, WA 98121, USA; Division of Pediatric Anesthesiology and Pain Medicine, Seattle Children’s Hospital, Seattle, WA, USA; Institute for Health Metrics and Evaluation, University of Washington, 2301 5th Ave Suite 600, Seattle, WA 98121, USA; Institute for Health Metrics and Evaluation, University of Washington, 2301 5th Ave Suite 600, Seattle, WA 98121, USA; Institute for Health Metrics and Evaluation, University of Washington, 2301 5th Ave Suite 600, Seattle, WA 98121, USA; Big Data Institute, Li Ka Shing Centre for Health Information and Discovery, University of Oxford, Oxford, UK

## Abstract

**Background:**

Diarrheal diseases are the third leading cause of morbidity and mortality in children under 5 in Africa, responsible for an estimated 30,000,000 (95% Uncertainty Interval [UI], 27,000,000 - 33,000,000) severe cases and 330,000 (95% UI, 270,000 - 380,000) deaths in 2015. Developing targeted approaches to combat this burden is hampered by the lack of comprehensive, fine-scale diarrhea estimates between and within countries.

**Methods:**

Annual estimates of diarrheal prevalence, incidence, and mortality were produced with high geographic detail (5-km2) across Africa from 2000 to 2015. Estimates were created using Bayesian geostatistical techniques, and were calibrated to the results from the Global Burden of Diseases, Injuries, and Risk Factors Study 2016.

**Results:**

The results revealed geographic inequality in diarrhea risk in Africa. Of the estimated 330,000 childhood deaths attributable to diarrhea in 2015, over 50% occurred in only 55 (of the 782) first administrative subdivisions. In 2015, mortality rates between first administrative subdivisions in Nigeria exhibited six-fold differences. The case fatality ratio is highly variable at the national level across Africa, with the highest values observed in Lesotho, Mali, Benin, and Nigeria.

**Conclusions:**

Our findings show concentrated areas of morbidity and mortality associated with diarrhea across countries with consistently high burden as well as countries that experienced considerable national-level improvements. In the era of precision public health, the distribution of limited resources can be optimized with proven interventions, targeted at locations most likely to have a high impact, reducing the avertable burden of diarrheal diseases.

## Introduction

Childhood diarrheal deaths are largely preventable. Unfortunately, the burden of diarrhea remains high and inadequately characterized due to the complex interplay that the environment, food, water, and sanitation have with poverty and deprivation.^1^ A significant proportion of cases can be prevented through rotavirus immunization,^2,3^ safe drinking-water,^4^ safely-managed sanitation and hygiene,^5^ and establishment of processes to eliminate exposure to contaminated food.^6^ Meanwhile, case management with oral rehydration salts (ORS),^7,8^ zinc supplementation,^9,10^ and antibiotics^11^ have the potential to prevent those with diarrhea from dying. Clear information on locations with the greatest diarrheal burden is required to accelerate progress and efficiently target intervention and treatment programs. 

The Global Burden of Diseases, Injuries, and Risk Factors Study 2016 (GBD 2016) estimates that 330,000 children under 5 – approximately 2 in 1,000 – died from diarrheal diseases in 2015 in Africa.^3^ Since 2000, the diarrhea mortality rate has decreased by 54% and the severe diarrhea incidence rate has decreased by 18%. However, in part due to population growth between 2000- 2015, the absolute number of severe diarrhea episodes has increased from 25,650,000 (95% Uncertainty Interval [UI], 23,162,000 - 28,084,000) to 29,761,000 (95% UI, 26,598,000 - 33,421,000).^12^ Because of this significant amenable mortality^13^ and the long-lasting negative health impacts on nutrition, growth, and development with recurrent diarrhea,^14,15^ further reduction of the global diarrhea burden remains a priority. 

Initiatives such as the Global Action Plan for the Prevention and Control of Pneumonia and Diarrhea (GAPPD) establish ambitious goals to address the high diarrhea burden among children. These goals aim to reduce child mortality rates to below 1 in 1,000 persons and reduce severe diarrhea episodes to 75% of their 2010 values by 2025. Precision public health, the use of high resolution data to guide tailored interventions, is necessary to identify the most vulnerable populations and better target lifesaving preventive and treatment measures.^16,17^

No previous study has attempted a comprehensive, sub-national analysis of diarrhea burden across any large region of Africa, although there have been several focused analyses of spatial and spatio-temporal variation in diarrheal burden within selected countries.^1,12,18–20^ A history of mapping malaria burden 21,22 combined with recent work in mapping under-5 mortality rates, child growth failure,^23^ and educational attainment^24^ has demonstrated the utility of household surveys for identifying local patterns of health across the continent and thereby identifying the greatest opportunities for impact. 

Here we present the first comprehensive, systematic analysis of local variation in diarrheal morbidity and mortality in children under 5 across Africa during the Millennium Development Goal era (2000-2015). Using Bayesian model-based geostatistics, 51,355 geolocated point level survey clusters and 2,524 small geolocated polygons, and existing GBD 2016 methods, we produce yearly, 5-km2 gridded estimates of diarrhea prevalence, incidence by severity, and mortality for children under 5, from 2000 through 2015 across Africa. 

## Methods

We compiled a database of 191 surveys from Africa that contained geocoded information corresponding to coordinates of 51,355 survey clusters and 2,524 subnational polygon boundaries. Survey clusters are the geographic unit in the sampling design from which households are randomly sampled—often a village, enumeration area, or census tract. For data that we could not match to specific survey clusters (e.g. GPS data was unavailable), we instead identified the smallest area/polygon and aggregated all observations within the unit to that level for modelling. Sources were excluded if they did not record period prevalence of diarrhea for every child in the home in the proceeding 2 – 4 weeks; if they did not include strata, primary sampling unit, and design weights for each participant; and if they did not include geographic information more specific than national (admin0) scale. We included datasets from the Demographic and Health Surveys (DHS), Multiple Indicator Cluster Surveys (MICS), as well as World Bank and country-specific surveys from 1998-2016.^25-28^ It was more probable that surveys not part of a larger series conducted independently would be excluded due to missing these criteria. Each source recorded period prevalence of diarrhea for every child in every home sampled over the preceding two to four weeks. Details on each data source for each country are provided in the Supplementary Appendix. 

Prevalence data was adjusted for season and converted from period prevalence to point prevalence as described in the GBD 2016 study.^29^ The resulting adjusted point prevalence data was modeled directly in a Bayesian model-based geostatistical framework described in detail elsewhere.^1,29^ Briefly, a spatially and temporally explicit hierarchical logistic regression model was fit to point prevalence of diarrhea. In this model, points that are closer together in space and time – and which have similar covariate patterns – are expected to have similar diarrheal prevalence. To reflect the social, structural, and environmental factors that may influence diarrheal prevalence, we assembled a collection of 27 covariates (Table S3). Posterior distributions of all model parameters and hyperparameters were estimated using R-INLA.^30,31^ Due to the spatial resolution of the main covariates, all predictions were made at the 5-km^2^ scale. After fitting the geospatial model, 1,000 draws (samples) were taken from the joint posterior distribution of diarrheal prevalence. Each draw contains a single possible diarrheal prevalence value for each 5-km^2^ location for each modeled year. 

The GBD 2016 study produced estimates of diarrhea prevalence, incidence, and mortality for every country in Africa for each year from 1990-2016.^29^ We combined our posterior distributions from above with the modeled results and diarrhea severity distributions from GBD 2016 in two stages. First, we maintained consistency with the GBD 2016 estimates by scaling our results such that these 5-km^2^ estimates of diarrhea prevalence – when aggregated and averaged to the national level by calculating a population-weighted mean – match the national level GBD estimates for each country and year. Second, we used the GBD 2016 ratios between incidence, prevalence, and mortality for every country-year to convert our prevalence estimates to corresponding estimates for mortality and incidence of severe diarrheal episodes. Draws of prevalence, incidence, and mortality were then summarized as mean estimates and Bayesian uncertainty intervals. Aggregated administrative subdivision estimates were also calculated at the draw level and then summarized as population-weighted means with uncertainty intervals. Annual case fatality ratios were calculated for each country by dividing the number of diarrhea deaths estimated by the GBD project by the corresponding number of incident cases.^32^ Model validation was conducted both in-sample and out-of-sample using several hold out methods. Additional details on model, estimation, and validation processes can be found in the Supplemental Appendix, sections 3.0 and 4.0. 

This study complies with the Gather for Accurate and Transparent Health Estimates Reporting (GATHER) recommendations (Table S1). All code used for these analyses will be available online upon publication at https://github.com. Given the continental scope and fine spatial scale of this work, additional results are provided in the Supplemental Appendix and will be made available upon publication on an online visualisation tool (http://vizhub.healthdata.org/lbd/diarrhea), which will be updated annually. 

## Results

### Diarrheal mortality

**Figure 1: Diarrhea mortality rates in children under 5 in 2000 and 2015:**
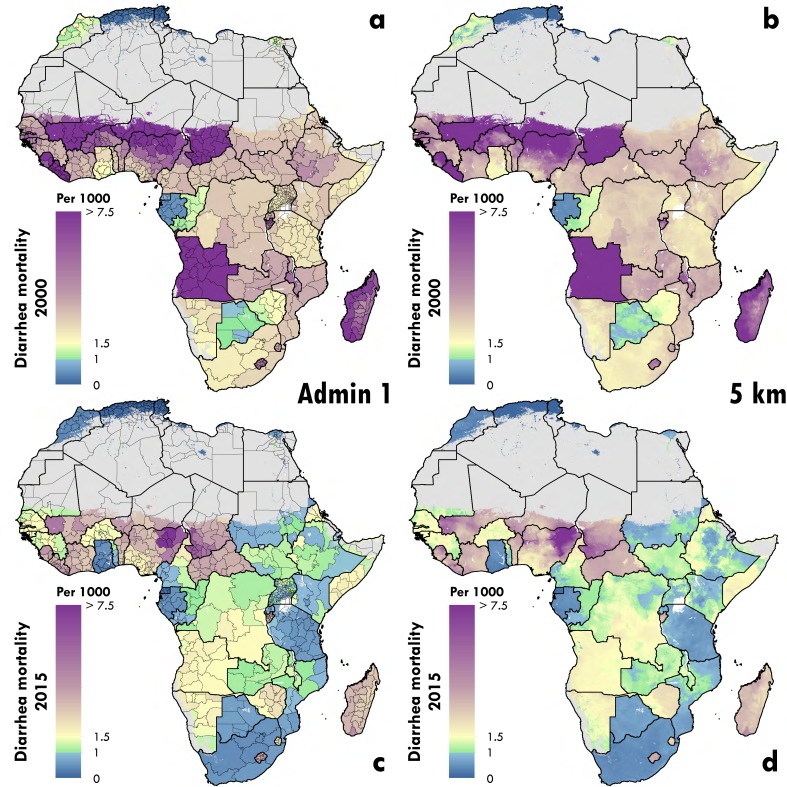
Panels A and B show the estimated mean rate per 1,000 of mortality attributable to diarrhea in 2000. Panels C and D show the estimated mean rate per 1,000 of mortality attributable to diarrhea in 2015. Panels B and D display the rates at the 5-km2 scale at which the model is fit. Panels A and C display the rates aggregated up to first administrative subdivision using population weighting. The color scales for mortality are set to indicate the locations in which the mean mortality rate estimates have achieved the GAPPD goal of less than 1 in 1,000. Pixels with fewer than ten people per 1-km2 and classified as “barren or sparsely vegetated” are colored in grey.

Our findings suggest an unequal distribution of diarrheal burden for children under 5 across Africa from 2000-2015. Locations in Nigeria and Chad have maintained high mortality rates through the study period; each country had several first administrative subdivision that exceed 6 deaths per 1,000 in 2015 (Figure 1). In 2015, the largest difference in within-country mortality rate observed was in Nigeria, with estimates ranging from Bayelsa at 1.6 (95% UI, 1.0 - 2.3) per 1,000 to Yobe at 9.5 (95% UI, 7.1 – 12.8) per 1,000 (Figure 1). 

**Figure 2: Ten highest number and rates of diarrhea associated mortality by first administrative subdivision from 2000 to 2015:**
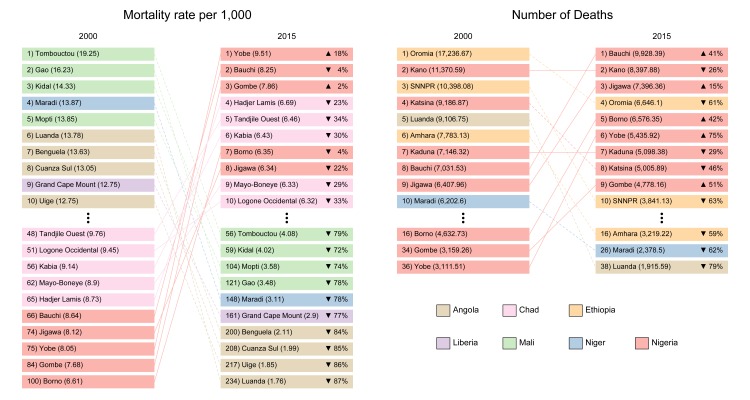
The left panel shows the 10 first administrative subdivisions with the most childhood death counts associated with diarrhea in 2000 and 2015. The right panel shows the 10 first administrative units with the highest mortality rates (per 1,000) associated with diarrhea in 2000 and 2015. Regions not in the top 10 in both 2000 and 2015 are listed below vertical ellipses with associated year-specific rank. The lines connecting regions are solid if rank increased from 2000 to 2015 and dashed if the rank decreased. Relative change in values is shown in the 2015 columns. SNNPR: Southern Nations, Nationalities, and People’s Region.

**Figure 3: Number of diarrheal deaths in children under 5 in 2000 and 2015:**
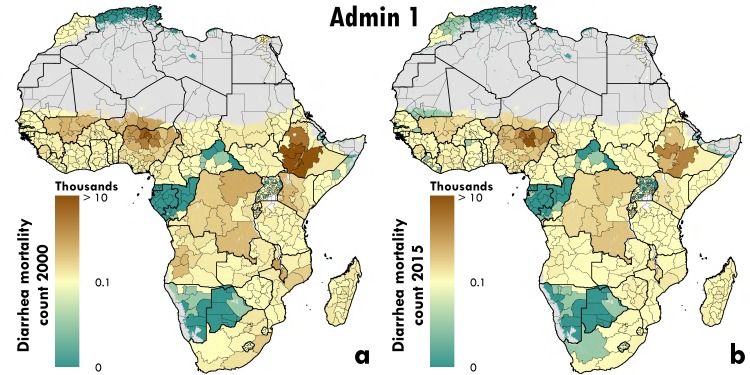
Panel A shows the estimated mean number of diarrheal death counts in 2000. Panel B shows the estimated mean number of diarrheal death counts in 2015. Both panels display diarrheal death counts aggregated up to the first administrative subdivision using population weighting. All color scales are on a log scale. Pixels with fewer than ten people per 1-km2 and classified as “barren or sparsely vegetated” are colored in grey.

Our estimates reveal that the number of under 5 deaths due to diarrhea in Africa is highly geographically concentrated. Bauchi, Gombe, and Yobe, the first administrative subdivisions with the three worst mortality rates in Nigeria, account for 6% of all diarrhea death count in Africa while making up just 1% of the population at risk (with 9,928 [95% UI, 7,583 – 13,019], 4,778 [95% UI, 3,515 – 6,494] and 5,436 [95% UI, 4,055 – 7,322] deaths, respectively) (Figure 2). Nearly 50% of all childhood diarrhea deaths in Africa were estimated to occur in just 7.0% (55/782) of the first administrative subdivisions on the continent (35% of population, Figure 3). While the burden of diarrheal deaths continues to vary across the continent, diarrheal mortality rates have decreased in nearly all locations in Africa from 2000 to 2015, increasing in only certain parts of the Central African Republic, Gabon, Zimbabwe, Côte d'Ivoire, and Nigeria (Figure 1). 

### Diarrheal incidence

**Figure 4: Severe diarrhea incidence rates in children under 5 in 2000 and 2015 in first administrative units:**
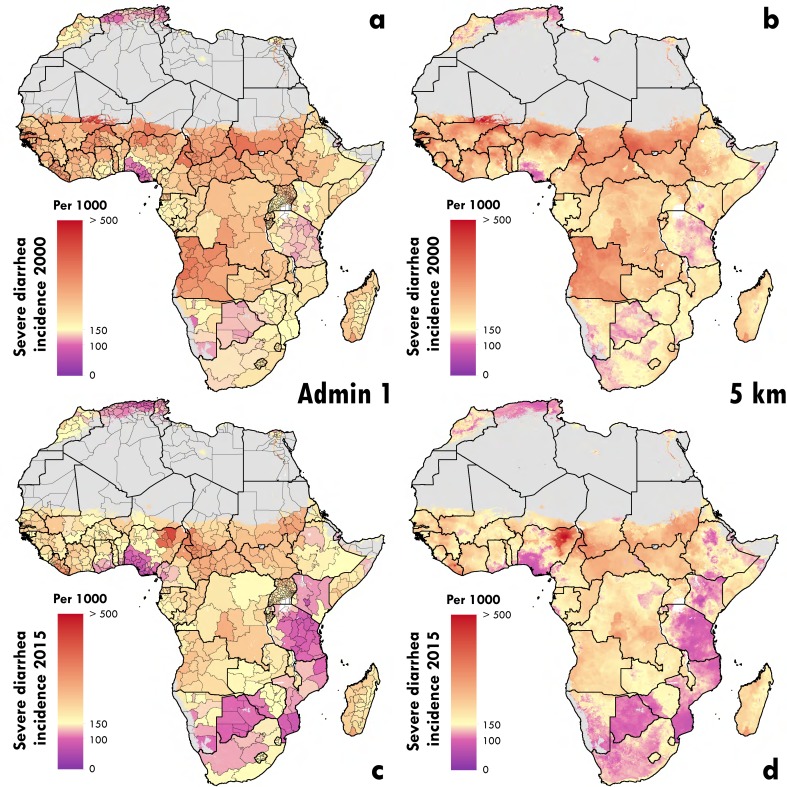
Panels A and B show the estimated mean rate per 1,000 of severe diarrhea episodes in 2000. Panels C and D show the estimated mean rate per 1,000 of severe diarrhea episodes in 2015. Panels B and D display the rates at the 5-km2 scale at which the model is fit. Panels A and C display the rates aggregated up to the first administrative subdivision using population weighting. Pixels with fewer than ten people per 1-km2 and classified as “barren or sparsely vegetated” are colored in grey.

Nigeria contains the regions with the highest rates of severe diarrhea cases per 1,000 in 2015 (Yobe, Bauchi, and Gombe at 422 (95% UI, 315 - 569), 366 (95% UI, 280 - 480), and 349 (95% UI, 257 – 474, respectively; Figure 4). The burden of diarrheal incidence was also highly concentrated within parts of Ethiopia and the Democratic Republic of the Congo (DRC). In 2015, 9.4% (2,800,000 [95% UI, 2,390,000 - 3,300,000]) of all severe cases of diarrhea in Africa took place within just five first-level administrative units in these two countries: the Southern Nations, Nationalities, and People’s Region (SNNPR), Oromia, and Amhara in Ethiopia and the Orientale and Katanga regions of the DRC. 

### Case fatality rates and avertable deaths

**Figure 5: Diarrhea CFR between 2000 and 2015 and deaths averted:**
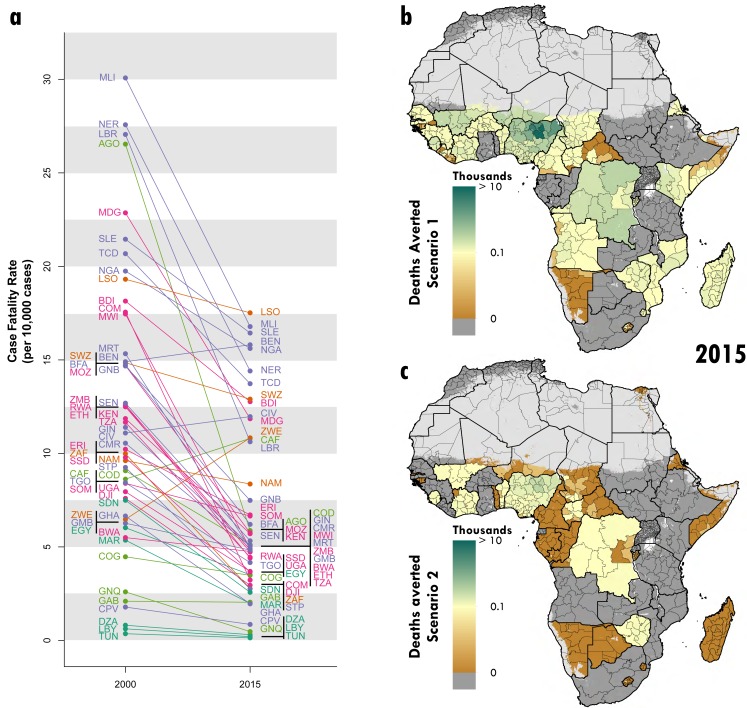
Panel A shows each country’s diarrheal CFR value in 2000 and in 2015. Panel B shows “Scenario 1,” the estimated number of deaths averted had all countries with the highest 50% CFRs in 2015 achieved the median CFR in 2015. Panel C shows, “Scenario 2,” the estimated number of deaths averted had the countries with the worst change in CFR between 2000-2015 achieved the median CFR change during that time period. Pixels with fewer than ten people per 1-km2 and classified as “barren or sparsely vegetated” are colored in grey.

In 2015, Lesotho (0.18% [95% UI, 0.12% - 0.25%]), Mali (0.17% [95% UI, 0.12% - 0.24%]), Sierra Leone (0.16% [95% UI, 0.11% - 0.23%]), Benin (0.16% [95% UI, 0.11% - 0.21%]), and Nigeria (0.16% [95% UI, 0.11% - 0.21%]) had the highest diarrheal case fatality rates in Africa (Figure 5 Panel A). Although the case fatality ratio in Benin increased from its estimated value in 2000 (0.15% [95% UI, 0.10% - 0.22%]), the remaining four countries listed above experienced relative improvements between 2000 and 2015. 

The median country-level case fatality ratio in 2015 was 0.0498%. Had all countries with case fatality ratios worse than the median 2015 value achieved the median 2015 case fatality ratio value (“Scenario 1”), an estimated 251,202 deaths (95% UI 220,859 – 283,164) could have been averted across the continent (Figure 5, Panel B). Approximately 41% of these averted deaths (103,161 [95% UI, 83,765 – 126,065]) would have occurred in Nigeria. Specifically, Bauchi (9,709 deaths averted [95% UI, 7,415 – 12,731]), Kano (8,212 deaths averted [95% UI, 5,987 – 11,228]), and Jigawa (7,232 deaths averted [95% UI, 5,307 – 9,703]) would have seen the most lives saved of any African first administrative subdivisions. 

Similarly, the median reduction in case fatality ratio from 2000 to 2015 was 51.4%, between that of Cameroon (51.0% [95% UI, 26.2% - 70.5%]) and Kenya (51.8% [95% UI, 42.8% - 59.7%]). If the countries below the median change during this period had reduced their case fatality ratio by this median value (“Scenario 2”), approximately 49,059 (95% UI, 41,888 – 56,314) lives could have been saved in 2015 (Figure 5, Panel C). 

### Data Validation

Validation of model fit and model specification were performed using two instances of 5-fold cross validation. Folds were spatially selected using a quad-tree algorithm or by second administrative unit, such that data near each other were selected for the same fold. This provided a more stringent test for our spatially correlated model, and more closely resembled the spatially patchy nature of data sparsity in the input data. Out of sample statistics such as root mean squared error (RMSE), correlation, and coverage were generated on the data held out of the model and subsequently summarized by aggregating to administrative areas. Across the continent at the first administrative unit level, we have an RMSE of .01039 for 2000 and 0.00962 in 2015, while the correlation for these years were 0.86 and 0.95 respectively. Additional statistics on model validity can be found in the supplementary materials. 

## Discussion

Our modeled maps demonstrate substantial local variation in both incidence and mortality associated with diarrhea in children under 5 in Africa over the last 15 years. The rates of decline in incidence and mortality have varied both between and within countries at every level of spatial aggregation considered. Some countries appear to have significantly reduced their diarrhea burden uniformly, while others are behind on their progress countrywide. Additionally, these high-resolution subnational estimates identify a third group of countries whose progress has varied subnationally. By providing estimates of current rates and counts of severe incidence and mortality, we identify locations most in need of interventions to reduce diarrhea burden.

Over half of all diarrheal deaths in Africa occur in about 7% of the first administrative subdivisions, which contain 35% of Africa’s population. These highly populated locations with high mortality rates - many of which are in Nigeria, Ethiopia, and Niger - are places where targeted interventions to improve mortality rate, even modestly, could avert many deaths. Conversely, in-depth evaluation of the factors contributing to success in countries like Ethiopia, where case fatality rate declined by over 60% from 2000 to 2015, could reveal important strategies for reducing case fatality in other areas. As noted in the work by Troeger et al.,^1^ Ethiopia has shown significant improvements in child nutrition over the last 15 years. That, combined with an expanded use in oral rehydration therapy, appears to account for much of the reduction of mortality in that country. 

The relative intractability of diarrhea incidence compared to diarrhea mortality, as shown in the present analysis and elsewhere,1 may suggest that growing access to timely and appropriate treatment, better nutritional status, and fewer comorbidities are contributing factors to reducing diarrhea mortality. A variety of interventions – including programs to promote immunization, hygiene, breastfeeding, oral rehydration therapy, and zinc supplementation – have been effectively employed on a small scale to combat diarrheal disease and death.^1,33^ Targeting the worst regions of those countries with the highest case fatality ratio, such as those in Lesotho and Mali, is likely to have a substantially larger impact than untargeted approaches. Though the introduction of the rotavirus vaccine into Africa is relatively recent and coverage is still incomplete, the GBD study found that rotavirus vaccine coverage was negatively correlated with all-cause diarrhea. There was however a significant range of estimated percent attributable fractions for rotavirus across Africa (6.5%-64.2% in 2016), and as the vaccine becomes more established this warrants further investigation. 

Local estimates of diarrheal burden can be used to prioritize improved access to safe water and sanitation, which varies greatly between dense and sparse populations;^34,35^ childhood growth monitoring, which has improved in most regions of Africa but not universally;^36,37^ delivery and uptake of vaccines, including the rotavirus vaccine;^38^ and access to diarrheal care and prevention interventions for marginalized populations that live in remote regions or areas of conflict.^39,40^ Nepal, for example, outpaced its neighboring countries in reducing diarrhea case fatality rates in part by implementing a district-level community intervention program.^41^ Additionally, Brazil successfully used targeted interventions in the 1980s, when it drastically reduced infant mortality due to diarrheal diseases through policy efforts aimed at the northeast of the country, a poorer region with the country’s highest burden.^42^

As with any work of this scope, our results are subject to several limitations. First, in order to produce continent-wide estimates, we combine data from a broad range of sources which require making assumptions about their utility and consistency. For example, while diarrheal prevalence was assessed with the same, standard question across heath surveys, they rely on self-reported stooling patterns, and as such are subject to recall and reporting bias. Additionally, conversions from prevalence to incidence leverage the GBD modeled estimates and the diarrhea severity distribution. Incorporating etiology-specific estimates of diarrheal incidence and severity would likely enhance the accuracy of the conversion. Similar to the GBD study which parses all-cause diarrhea into percent attributable fractions for multiple etiologies,1,29 we are working towards etiology-specific maps of mortality and morbidity for Africa. Currently neither approach uses information on bloody stools to assist in either severity splits or etiology-splits as that information was not included in all surveys. While the conversion from incidence to mortality leverages various data sources^1,12,29^ and allows for variation in case-fatality ratio by country, year, sex, and age, it does not currently allow for variation in case-fatality ratios by diarrheal etiology and does not incorporate the effects of comorbidities. Our geospatial approach naturally borrows strength from neighboring areas, and as such may smooth over extremely focal epidemics, such as those frequently associated with cholera. Finally, there is significant evidence of difference in risk within the 0-5 age group. Due to the nature of the data and methods we utilize, we are currently unable to parse mortality and morbidity estimates into finer age groups. 

This work provides a foundation for several important directions for future research. First, accounting for etiological distributions within prevalence, incidence, and death associated with diarrhea will provide increased capacity to create targeted intervention strategies (e.g. rotavirus vaccine coverage needs). Second, the approaches outlined in this work are directly applicable to other continents where similar data sources are available. Expanding estimates out of Africa to all low- and middle- income countries will be the next step towards the ultimate goal of globally mapping diarrheal morbidity and mortality. Third, as this statistical modeling approach deliberately values predictive performance over interpretability of the relationships between covariates and diarrhea, a parallel effort is underway to build spatio-temporal models more capable of causal inference to assess the impact of interventions such as vaccination and improvements in water, sanitation, and hygiene. These sorts of associations will be very important to explore in identifying the root causes of entrenched disease burden at the local- level. Future analyses will leverage these estimates to explore the extent to which high diarrheal burden in a subnational location reveals deeper patterns of eco-social inequity within countries. This work clearly demonstrates the marked local variability in childhood morbidity and mortality due to diarrhea across Africa. For every country in Africa, these estimates can be used to identify the optimal regions to more precisely target interventions. These estimated deaths are largely preventable at the population and clinical levels. Our work can help accelerate the already impressive reduction in childhood diarrhea deaths across the continent. 

## Supplementary Appendix

Supplementary Material
