# Supplementary material for: Local variation in childhood diarrheal morbidity and mortality in Africa, 2000-2015
Source: N Engl J Med. Author manuscript; Available in PMC 2018 Sep 20. (PMC6078160; doi:10.1056/NEJMoa1716766)
Supplement: Supplementary Material [file NEJMoa1716766_Reiner_Supplement.pdf]

Supplementary Appendix

1.0 Supplementary Discussion **2**
1.1 Incidence Rates of Change 2
1.2 Mortality Rates of Change 2
1.3 Mortality Posterior Probability of GAPPD Goal Achievement 2
1.4 Limitations 3
2.0 Data Sources **4**
3.0 Supplementary Methods **5**
3.1 Survey cluster combination and spatial integration over polygon records 5
3.2 Geostatistical model 5
3.2.1 Model geographies *5*
3.2.2 Ensemble covariate modelling *6*
3.2.3 Model Description *7*
3.2.4 Priors *8*
3.2.5 Mesh construction *8*
3.2.6 Model fitting and estimate generation *9*
3.2.7 Model Results *9*
4.0 Model Validation **10**
4.1 In-sample metrics 10
4.2 Metrics of predictive validity 10
4.3 Post estimation calibration to national estimates 10
5.0 Supplementary Figures **12**
6.0 Supplementary Tables **33**
7.0 Supplementary Appendix References **51**

1.0 Supplementary Discussion

1.1 Incidence Rates of Change

In contrast to the declines observed in diarrheal mortality, there were many regions with negligible decreases or even increases in diarrheal cases from 2000 to 2015. The calculated annualized rates of change (AROC) for incidence of severe diarrhea cases shows low to moderate gains (**Figure S1, Panel A**), and reductions exceeding 5% per year were rare [p<.05]. As illustrated by the direct comparison between 2000 and 2015 severe incidence rates, 118 first administrative subdivisions demonstrated an increase in severe incidence rate (**Figure S1, Panel A**).

1.2 Mortality Rates of Change

Average annual rates of change for mortality rates have exceeded 7.5% per year across much of Africa (**Figure S2, Panel A and B**). Across Sahelian regions with high mortality rates in 2000, many locations in Western sub-Saharan Africa have significant decline in mortality (e.g., Ghana, Senegal, and Liberia). Conversely, gains in regions of some of the worst Sahelian countries has been limited (Niger, Nigeria, Chad, Central African Republic, South Sudan, and Somalia). In central and southern Africa, there have been limited gains in Zimbabwe and Namibia.

1.3 Mortality Posterior Probability of GAPPD Goal Achievement

Using posterior draws for 2015 to assess progress towards the GAPPD mortality goal of less than 1 in 1,000 deaths attributable to diarrhea, we find that multiple countries have already surpassed this threshold (**Figure S2, Panel C and D**). Countries that are estimated to be close to the goal may or may not have correspondingly high or low posterior probabilities of achieving the goal as the probability incorporates uncertainty in the estimates. For example, within Mozambique across first administrative subdivisions, mean mortality rates vary from 0.8 to 1.1 per 1,000 in 2015. However, due to non-symmetric uncertainty, all posterior probabilities that the true value is less than 1 in 1,000 are greater than 30%.

Several countries began the millennium with such a high mortality rate – either uniformly or within subdivisions – that, despite substantial gains, results in the regions' mortality remaining well above the GAPPD target for diarrheal mortality. Cameroon, Rwanda, and much of Western sub-Saharan Africa have subnational mean mortality AROCs of 5% or greater (**Figure S2, Panels A and B**), but due to their high initial mortality rates (**Figure 1, Panel A and B**), estimated mortality remains high in 2015 (**Figure 1, Panels C and D**) and the probability they have achieved the GAPPD goal is very low (**Figure S2, Panels C and D**). As with other countries with extremely high mortality rates in 2010, even with the substantial gains made on the western portion of the border between Niger and Nigeria, the posterior probability that they have already met the mortality goal there is very low.

Using the calculated AROCs (either at the pixel level or calculated on the aggregated first administrative subdivision level), projections of GAPPD mortality goal attainment can be estimated (**Figure S2, Panels E and F**). In 2015, many locations that had achieved great gains in reduction of mortality were still above the established goals (**Figure S2, Panels C and D**). Conversely, given 10 more years of similar relative reductions in mortality the picture changes substantially (**Figure S2, Panels E and F**). With steady decline in mortality, DRC appears on track to reduce mortality well below the 1 in 1,000 threshold, along with Ethiopia, Benin, Togo, and much of Angola. South western Nigeria is projected to have pockets that are very likely to have achieved the mortality goal. Northern Cameroon started 2000 with rates comparable to both neighboring Chad and Nigeria, but due to significant declines in mortality risk, they are nearing the mortality goal.

1.4 Limitations

Our analysis assumes that the same proportion of incident cases are severe across all countries and years. This lack of spatio-temporal variation in severity over time prohibits our 2025 projection analysis from definitively concluding that this goal is unattainable. If diarrhea incidence is proportional to severe diarrhea incidence, as we have assumed in this analysis, it is unlikely that the majority of Africa will achieve a 75% reduction from the 2010 baseline by 2015.

2.0 Data Sources

The data sources used to model diarrhea prevalence indicators are described below. Information on survey locations, years, source, polygons, and/or geopositioned survey clusters can be found in **Table S2.** Data availability for each indicator can be found in **Figure S2.**

Select data sources that were identified to contain diarrhea prevalence within the geographic area of interest were excluded for the following reasons: missing survey weights for areal data, incomplete sampling (e.g., only a specific age range), or untrustworthy data (as determined by the survey administrator or by inspection). Within each source, administrative units with a sample size of one were excluded.

3.0 Supplementary Methods

3.1 Survey cluster combination and spatial integration over polygon records

Our individual-level data were collapsed (summarized) into clusters if they could be georeferenced to latitude-longitude pairs. Otherwise, we collapsed our individual-level data to the smallest polygon that could be referenced. We used survey weights and the *survey* package in R to account for matching our observations to a higher resolution than the representative resolution of the survey.[1]

Data without latitude and longitude, but that could be geolocated to an administrative area, were resampled to generate candidate point locations based on the underlying population of the administrative area. The main concept is to leverage covariate values across the polygon when performing the regression, while simultaneously accounting for a population-driven survey design. The methods used for the resampling are consistent with those used in geospatial modelling of under-5 mortality, published previously.[2]

For each polygon-level observation, 10,000 points were randomly sampled from within the polygon (regardless of the polygon's area) using the WorldPop total population raster[3] to weight the locations of the draws. K-means clustering was performed on the candidate points to generate integration points (1 per 1,000 pixels) used in the modelling. Weights were assigned to each integration point proportionally to the number of candidate points that entered into the k-means cluster, such that the weight of each point represented the number of population-sampled locations contained within the K-means cluster location, divided by the number of sampled points generated (10,000). Each point generated by this process is assigned the diarrhea prevalence observed from the survey for that polygon. These sample weights are used in model fit.[4]

3.2 Geostatistical model

3.2.1 Model geographies

A total of five models were run for each indicator based on continuous geographic regions within Africa chosen to align with the regions used in the Global Burden of Disease Study, which determines regions based on both proximity and epidemiological similarity (see **Figure S3** for listing of regions and countries). Minor changes were made to the GBD regions to ensure spatial contiguity across Africa. Initial investigation of regional configurations identified both the Democratic Republic of Congo and Egypt as outliers in their respective regions. As such, each was fit as individual regions, see **Figure S5** for an illustration of the modelling regions. All data within the spatial region, and within a one-degree buffer from the boundaries of each region, were included in each model to minimize edge effects.

As this study was limited to mainland Africa and African island nations, select countries were excluded from the North Africa and Middle East region (Afghanistan, Bahrain, Iran, Iraq, Jordan, Kuwait, Lebanon, Oman, Palestine, Qatar, Saudi Arabia, Syria, Turkey, UAE, and Yemen). Western Sahara was included as part of the North region. Several countries were

moved to East (Lesotho and Swaziland from South, Sudan from North) to make high-income status more similar in the North and South regions.

3.2.2 Ensemble covariate modelling

An ensemble covariate modelling method was implemented in order to both select covariates and capture possible non-linear effects and complex interactions between them.[5] For each region, four sub-models were fit to our dataset, using all of our covariate data as explanatory predictors: generalised additive models, boosted regression trees, lasso regression, and ridge regression. Sample weights are used in sub-models, where applicable, such that survey cluster locations with latitude and longitude had a sample weight of 1, while survey cluster locations where the latitude and longitude was generated by the polygon resampling process had a weight based on the K-means clustering process (refer to **section 3.1**).

Each sub-model is fit using five-fold cross-validation to avoid overfitting. The out-of-sample predictions from across the five holdouts are compiled into a single comprehensive set of predictions from that model. Additionally, the same sub-models were also run using 100% of the data, and a full set of in-sample predictions were created. The five sets of out-of-sample sub-model predictions are fed into the full geostatistical model as the explanatory covariates when performing the model fit. The in-sample predictions from the sub-models are used as the covariates when generating predictions using the fitted full geostatistical model. A recent study has shown that this ensemble approach can improve predictive validity by up to 25% over an individual model.[5]

Predictions from each sub-model are generated based on patterns and relationships between the raw covariates and prevalence data, while predictions from the full geostatistical model are generated based on patterns and relationships between the predictions from the ensemble of sub-models and prevalence data. To discover the relationships between the sub-model prediction layers (used as covariates in the full geostatistical model) and the prevalence data, the only values of the covariates (sub-model prediction layers) “seen” by the model are the values underlying the locations of surveys. As such, it is possible that estimates will be generated in areas where the values of the covariates exceed the minimum and maximum values observed by the model. In these areas, the estimates are generated by extrapolating from the patterns observed within the range of covariates underlying the survey and census data.

3.2.3 Model Description

Binomial count data are modelled within a Bayesian hierarchical modelling framework using a logit link function and a spatially and temporally explicit hierarchical generalised linear regression model to fit prevalence of each of our indicators in seven regions of Africa as illustrated in **Figure S5**.[6] For each GBD region, we explicitly write the hierarchy that defines our Bayesian model as follows:

$$C_i | p_i,\ N_i \sim Binomial(p_i,\ N_i)$$

$$logit(p_i) = \beta_0 + \boldsymbol{X_i \beta} + \epsilon_{GP_i} + \epsilon_{ctry_i} + \epsilon_i$$

$$\sum \boldsymbol{\beta} = 1$$

$$\epsilon_{ctry_i} \sim N(0, \sigma^2_{ctry})$$

$$\epsilon_i \sim N(0, \sigma^2_{ug})$$

$$\epsilon_{GP} | \Sigma_{\text{space}}, \Sigma_{time} \sim GP(0,\ \Sigma_{\text{space}} \otimes \Sigma_{time})$$

$$\Sigma_{\text{space}} = \frac{2^{1-\nu}}{\tau \times \Gamma(\nu)} \times (\kappa \boldsymbol{D})^{\nu} \times \mathrm{K}_{\nu}(\kappa \boldsymbol{D})$$

$$\Sigma_{time_{j,k}} = \rho^{|\, t_k - t_j|}.$$

For each geospatial risk factor and region, we model the number of children at survey cluster$i$, among a sample size, N_i, who are afflicted with a risk factor as binomial count data, C_i. We have suppressed the notation, but the counts, C_i, probabilities, p_i, predictions from the five submodels $\boldsymbol{X_i}$, and residual terms ϵ_* are all indexed at a space-time coordinate. The probabilities, p_i represent both the annual prevalence at the space-time location and the probability that an individual child will be afflicted with the risk factor given that they live at that particular location. The logit of annual prevalence, p_i, of our indicators was modelled as a linear combination of the three sub-models (generalized Additive Model , boosted regression tree, elastic net penalized regression), $\boldsymbol{X_{i,}}$a correlated spatio-temporal error term, ϵ_{GP_i}, and an independent error term, ϵ_i. Coefficients, $\boldsymbol{\beta}$, on the sub-models represent their respective predictive weighting in the mean logit link and are constrained to sum to one. The joint error term, ϵ_{GP}, accounts for residual spatio-temporal autocorrelation between individual data points that remains after accounting for the predictive effect of the sub-model covariates, ϵ_{ctry_i} is a

country random effect, and ϵ_i, which is an independent error term. The residuals, ϵ_{GP}, are modelled as a three-dimensional Gaussian process in space-time centered at zero and with a covariance matrix constructed from a Kroenecker product of spatial and temporal covariance kernels. The spatial covariance, Σ_{space}, is modelled using an isotropic and stationary Matérn function,[7] and temporal covariance, Σ_{time}, as an annual autoregressive order 1 (AR1) function over the 16 years represented in the model. This approach leveraged the data's residual correlation structure to more accurately predict prevalence estimates for locations with no data, while also propagating the dependence in the data through to uncertainty estimates.[8] The posterior distributions were fit using computationally efficient and accurate approximations in R-INLA[9,10] (integrated nested Laplace approximation) with the stochastic partial differential equations (SPDE)[11] approximation to the Gaussian process residuals.

3.2.4 Priors

The following priors were used for all four of our diarrhea models:

- $\beta_0 \sim N(\mu = 0, \sigma^2 = 1000)$,
- $\boldsymbol{\beta} \sim^{iid} N\left(\mu = \frac{1}{\text{\# ensemble models}}, \sigma^2 = 1000\right)$,
- $log\left(\frac{1+\rho}{1-\rho}\right) \sim N(\mu = 0, \sigma^2 = 1/0.15)$,
- $log\left(\frac{1}{\sigma_{ctry}^2}\right) \sim loggamma(\alpha = 1, \gamma = 2)$,
- $log\left(\frac{1}{\sigma_{nug}^2}\right) \sim loggamma(\alpha = 1, \gamma = 2)$.
- $\theta_1 = \log(\tau) \sim N(\mu_{\theta_1}, \sigma_{\theta_1}^2)$
- $\theta_2 = \log(\kappa) \sim N(\mu_2, \sigma_{\theta_2}^2)$

We used the uncorrelated multivariate normal priors that INLA automatically determines (based on the finite elements mesh) for the log-transformed spatial hyperparameters κ and τ. The mean (μ) and variance (σ^2) parameters for the hyperpriors selected by INLA for the meshes in each region can be found in **Table S4**. In our parameterization we represent α and γ in the *loggamma* distribution as scale and shape, respectively.

3.2.5 Mesh construction

We constructed the finite elements mesh for the stochastic partial differential equation approximation to the Gaussian process regression using a simplified polygon boundary (in which coastlines and complex boundaries were smoothed) for each of the regions within our model. We set the inner mesh triangle maximum edge length (the mesh size for areas over land) to be 0.2 degrees, and the buffer maximum edge length (the mesh size for areas over the ocean) to be 5.0 degrees. An example finite elements mesh constructed for central sub-Saharan mesh can be found in **Figure S6**.

3.2.6 Model fitting and estimate generation

Models were fit in INLA with methods consistent with those used in geospatial modelling of under-5 mortality, published previously.[2]

Resampling K-means weights (**refer to section 3.2**) were used within the INLA fit by multiplying the corresponding log-likelihood evaluation for the specific observation by the observation's K-means weight. Data points that could be georeferenced to latitude-longitude locations were assigned a weight of 1, ensuring that when the log-likelihood contribution from that observation was evaluated it contributed only to the log-likelihood at the observation's space-time location. For survey cluster locations generated based on the polygon resampling process, the log-likelihood of those points contributed proportionate to the K-means weights, effectively diffusing the evaluation of the observation across the polygon.

As part of the ensemble modelling process (**section 3.3.2)**, prediction surfaces from the out-of-sample ensemble sub-models were used as covariates in the spatio-temporal model. Estimates of the fixed effects beta coefficients derived from the contribution of each of the sub-models to INLA's predicted prevalence estimates, in conjunction with parameter estimates of the contribution of location and time (based on estimated parameters described in model description (**refer to section 3.3.3**) were generated and can be found in **Table S5**. To create final estimates, the in-sample prediction surfaces of prevalence from the sub-models (serving as covariates) were used to calculate estimates of prevalence for each pixel in each year.

All estimates were generated by taking 1,000 draws from the posterior distribution. For estimates at the pixel level, these draws were used directly to generate estimates and uncertainty. Aggregated estimates, in which estimates at the pixel level were summarized to administrative boundaries, were generated by creating population-weighted averages for each administrative boundary, for each draw. 95% uncertainty intervals around the mean of our estimates (**Figure S7**) were generated by taking the 2.5% and 97.5% quantiles of each of the draws, at the pixel or administrative level.

3.2.7 Model Results

Fitted parameters and hyperparameters, as well as their 95% uncertainty intervals are shown by indicator and region in **Table S5**. Spatial hyperparameters (τ and κ) and their uncertainties have been transformed into more interpretable nominal variance and range parameters. Nominal variance, approximating the variance at any single point, is calculated as $nom.var = 4\pi\kappa^2\tau^2$, and nominal range, approximating the distance before spatial correlation decays by 90%, as $range = \sqrt{8}/\kappa$.

4.0 Model Validation

4.1 In-sample metrics

For each indicator, we generated a suite of diagnostic plots for each region and country estimated, in order to assess the in-sample performance of our model and compare to national-level estimates produced by GBD.

To explore residual error over space and time, absolute error (data minus predicted posterior mean estimates at the corresponding pixels) were produced at five-year intervals (2000, 2005, 2010, and 2015) for each modelled region (**Figure S8**).

4.2 Metrics of predictive validity

In order to assess the predictive validity of our estimates, we validated our models using spatially stratified five-fold out-of-sample cross-validation.[12] We used a modified bi-tree algorithm that recursively partitions two-dimensional space, alternating between horizontal and vertical splits on the weighted data sample size medians, until the data contained within each spatial partition are of a similar sample size. The depth of recursive partitioning is modulated by the target sample size (400) within a partition and the minimum number of survey clusters or pseudo-clusters allowed within each spatial partition (in this case, a minimum sample size of 125 was used). These spatial partitions are then allocated to one of five folds for cross-validation. As an additional test, we used the second administrative subdivisions to define spatial our holdouts. Results from both strategies can be seen in Figures S9 -S11 and Tables S6 - S9.

For validation, each geostatistical model was run five times, each time holding out data from one of the folds, generating a set of out-of-sample predictions for the held-out data. For each indicator, a full suite of out-of-sample predictions over the entire dataset was generated by combing the out of sample predictions from the five cross-validation runs.

Using these out-of-sample predictions, we then calculated mean error (ME, or bias), root-mean-squared-error (RMSE, which summarizes total variance), and 95% coverage of our predictive intervals (the proportion of observed out-of-sample data that fall within our predicted 95% uncertainty intervals) aggregated up to different administrative levels (levels 0, 1, and 2) as defined by FAO Global Administrative Unit Layers (GAUL).[13]

4.3 Post estimation calibration to national estimates

In order to leverage national-level data included in GBD 2016, but outside the scope of our current geospatial modelling framework (which requires point or relatively small unit administrative level data), and to ensure perfect calibration between these estimates and GBD 2016 national-level estimates, we performed a post hoc calibration such that the population weighted mean of the draws recovers the mean estimate from the GBD.[14]

Specifically, for each country-year, we find a value k such that the $\sum invlogit(logit(p_i) + k) * pop$ equals the number of prevalent cases reported in GBD, where p_i is the predicted prevalence at a given x/y coordinate, and pop is the population of all children under five at that same coordinate. The summation occurs over all coordinates within the selected country-year. To allow comparison between our modelled estimates and the GBD 2016[14] national-level estimates to which they were calibrated, **Figure S12** plots the mean uncalibrated estimates from the model-based geostatistics (MBG) process aggregated to the national-level ("MBG mean") as compared to the GBD national estimates ("GBD mean") for all modelled years.

Estimates produced by MBG models were also compared to raw estimates from the DHS series, each aggregated to the first subnational geographic subdivision. These results can be found in **Figure S13**.

5.0 Supplementary Figures

Figure S 1. Diarrhea incidence annualized rate of change and posterior probability of meeting GAPPD goal for 2025 13
Figure S 2. Diarrhea mortality annualized rate of change and posterior probability of meeting GAPPD goal for 2015 and 2025 14
Figure S 3. Diarrhea data availability by type and country, 2000–2015 15
Figure S 4. Covariates 16
Figure S 5. Map of modelling regions 17
Figure S 6. Finite elements mesh 18
Figure S 7. Posterior means and 95% uncertainty intervals 19
Figure S 8. Plots of Diarrhea prevalence absolute error in Africa 19
Figure S 9. Admin 0 aggregation 21
Figure S 10. First administrative subdivision aggregation 23
Figure S 11. Second administrative subdivision aggregation 25
Figure S 12. Comparison of aggregated MBG estimates to GBD 2016 diarrhea prevalence estimates 27
Figure S 13. First administrative subdivision Comparison of MBG estimates to DHS estimates 28
Figure S 14. Out of Sample Statistics of different model specifications 29
Figure S 15. Geospatial Modeling Flowchart 31

Figure S 1. Diarrhea incidence annualized rate of change and posterior probability of meeting GAPPD goal for 2025

Panel A shows the estimated mean annualized decrease in severe diarrhea incidence from 2000 to 2015. Panel B shows the posterior probability that the projected 2025 rates of severe diarrhea incidence reach 75% of the severe incidence rate in 2010. Pixels with fewer than ten people per 1-km^2 and classified as “barren or sparsely vegetated” are colored in grey.

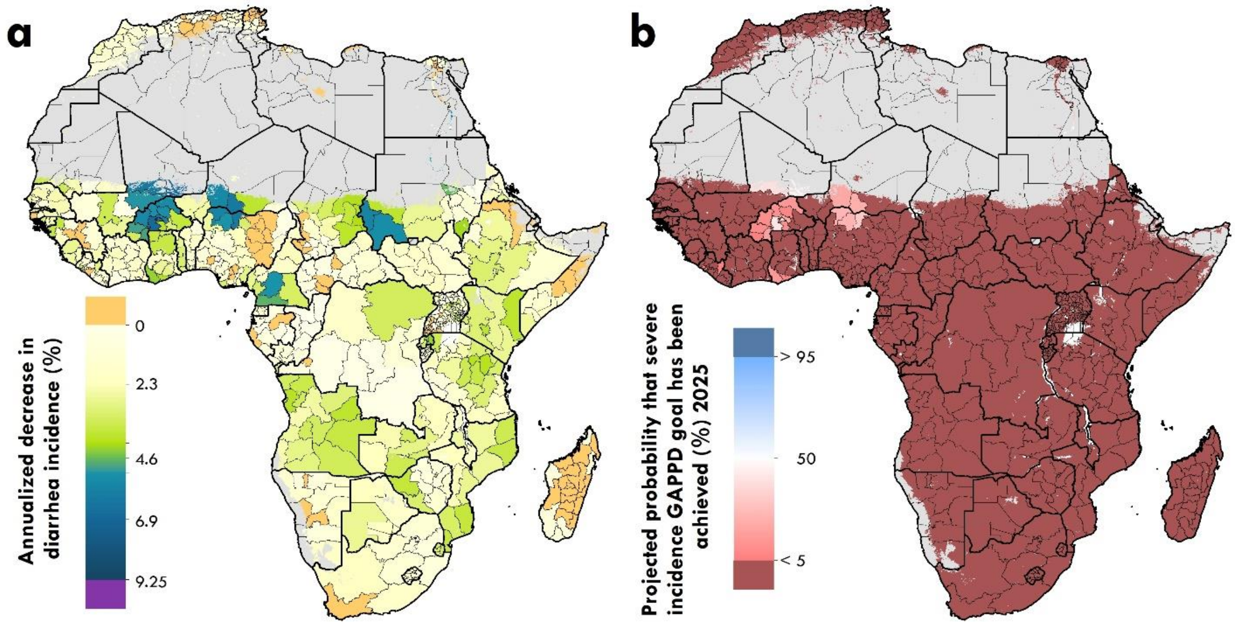

Figure S 2. Diarrhea mortality annualized rate of change and posterior probability of meeting GAPPD goal for 2015 and 2025

Panel A and B show estimated mean annualized decrease in diarrhea mortality from 2000 to 2015. Panel C and D show the posterior probability that the 2015 rate of mortality attributable to diarrhea is less than 1 in 1,000. Panel E and F show the posterior probability that the projected 2025 rates of mortality attributable to diarrhea are less than 1 in 1,000. Panels B, D, F display the rates at the 5-km^2 scale at which the model is fit. Panels A and C display the rates aggregated up to the first administrative subdivision using population weighting. Pixels with fewer than ten people per 1-km^2 and classified as "barren or sparsely vegetated" are colored in grey.

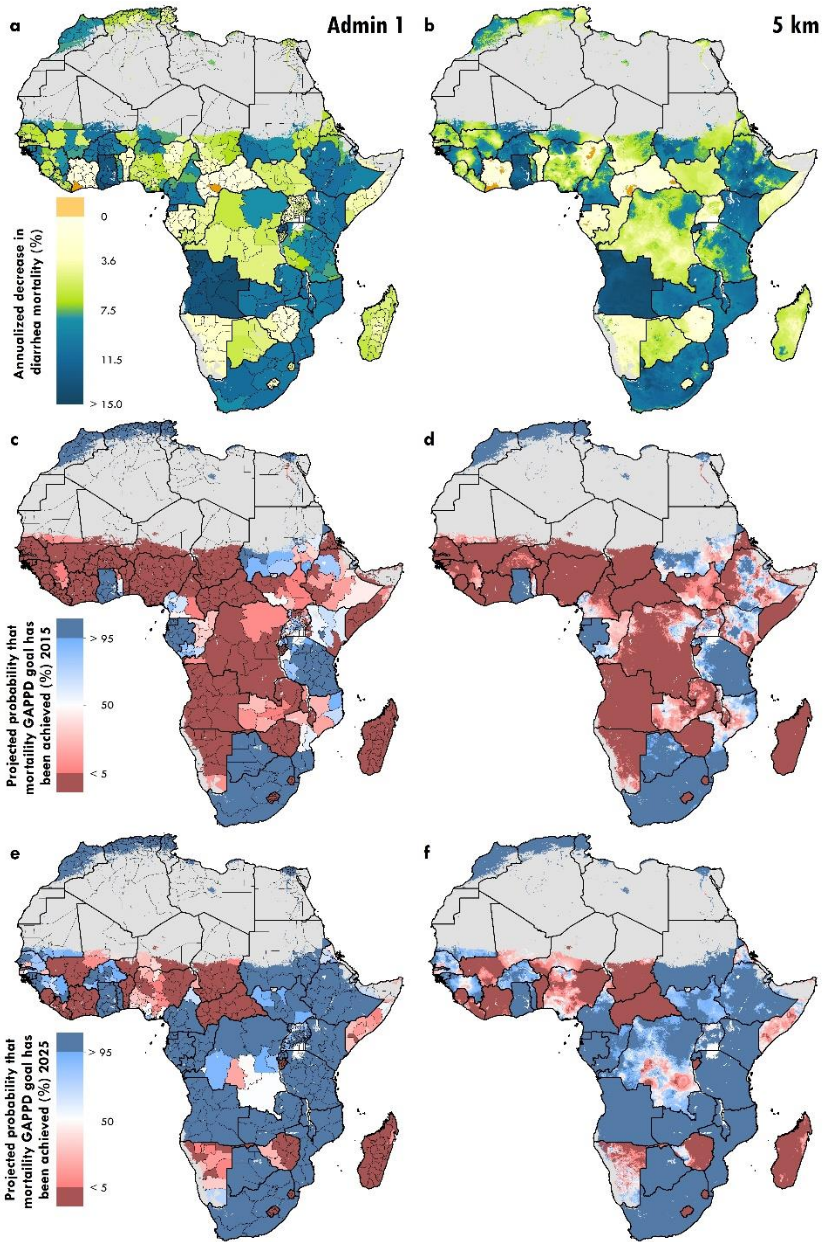

Figure S 3. Diarrhea data availability by type and country, 2000–2015

All data are shown by country and year of survey and are mapped at their corresponding geopositioned coordinate or area. The total number of points and polygons (areal) for each country are plotted by data source, type, and sample size (left side). Sample size represents the number of individual microdata records for each survey. Diarrhea prevalence for the input coordinate or area are mapped (right side). This database consists of 51,355 survey clusters and 2,524 polygons.

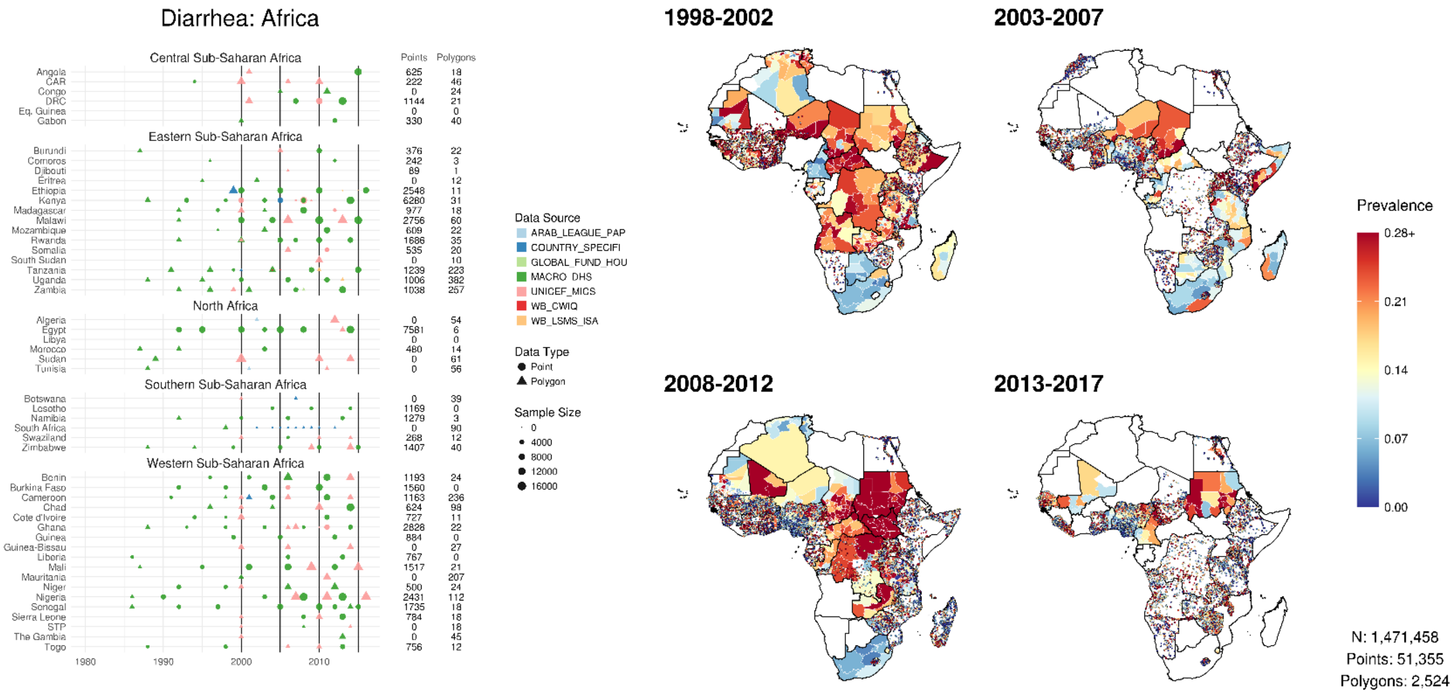

Figure S 4. Covariates

Twenty-nine covariate raster layers of possible socioeconomic and environmental correlates of diarrhea prevalence in Africa were used as inputs for the stacking modelling process. Time-varying covariates are presented for the year 2015. National level GBD covariates not included in this figure are: lag distributed income per capita, percent of population with access to improved toilet types, log-transformed SEV scalar for Diarrhea, and SEV unsafe water. For the year of production of non-time-varying covariates, please refer to the individual covariate citation **Table S 3** for additional detail. Pixels with fewer than ten people per 1-km^2 and classified as "barren or sparsely vegetated" are colored in grey.

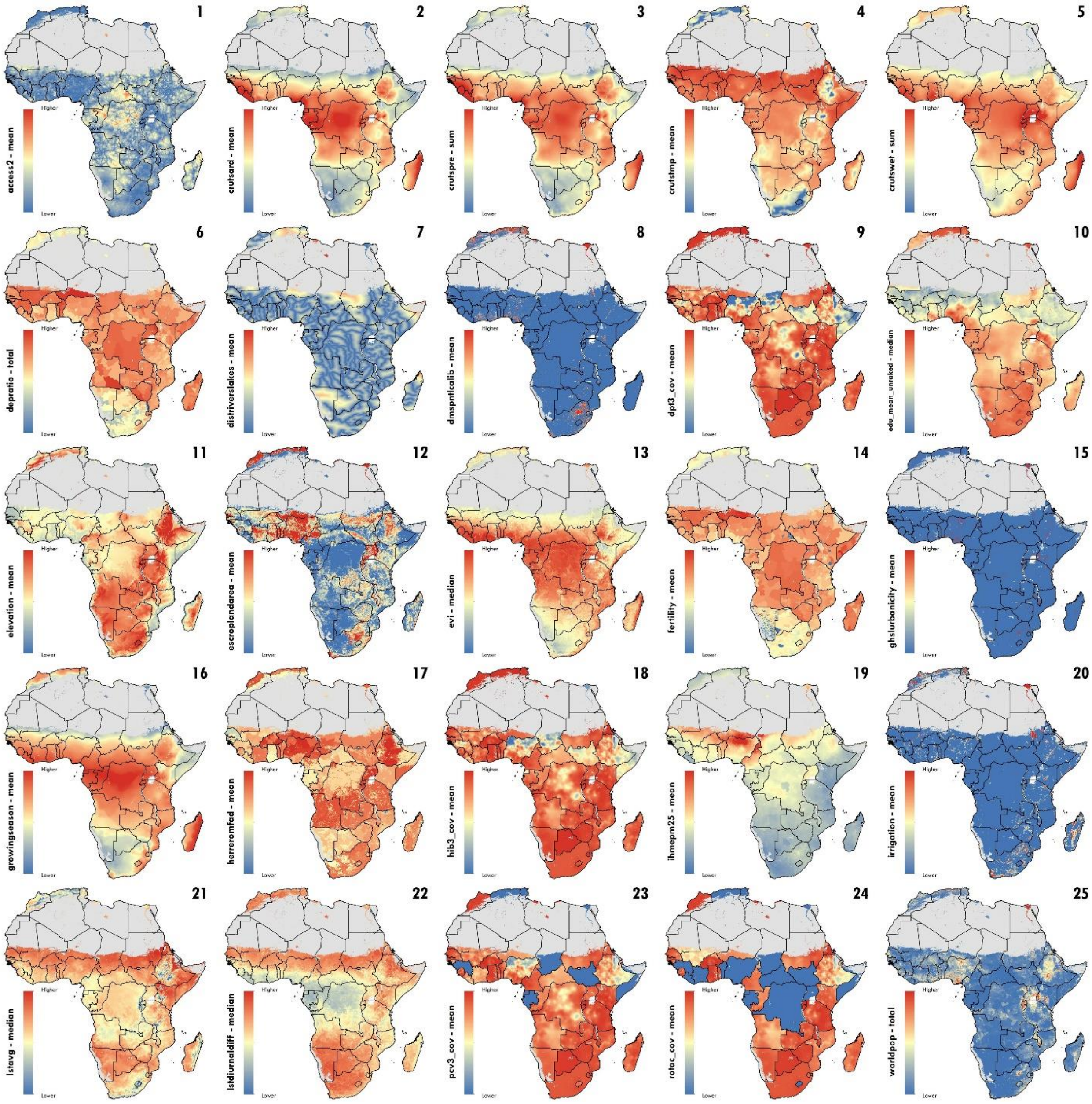

Figure S 5. Map of modelling regions

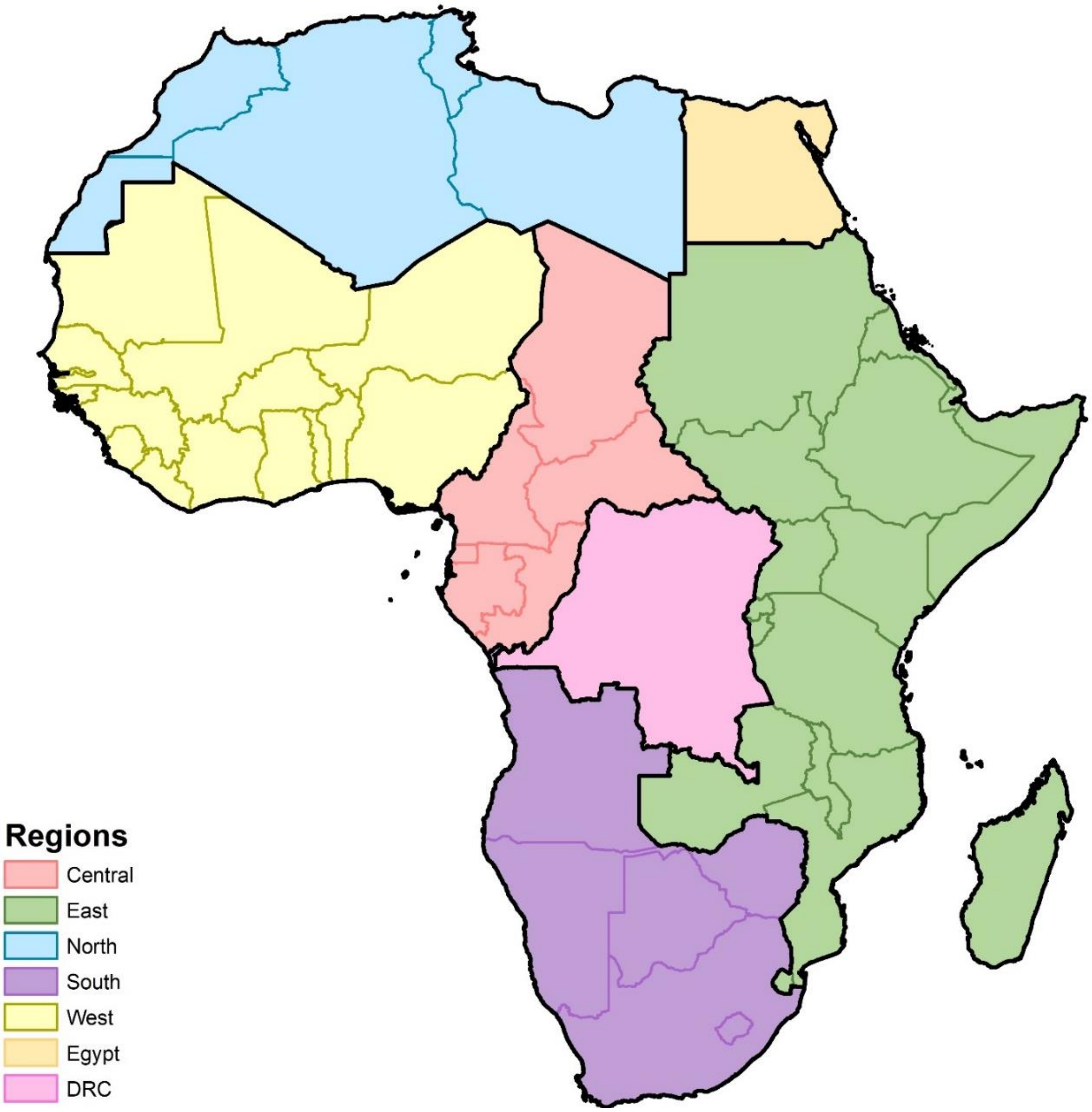

Figure S 6. Finite elements mesh

The finite elements mesh used to fit the space-time correlated error for the Central Africa region. Both the fine-scale mesh over land in the modelling region and the coarser buffer region mesh are shown. The simplified region polygon used to determine the boundary for the modelling region is shown in blue.

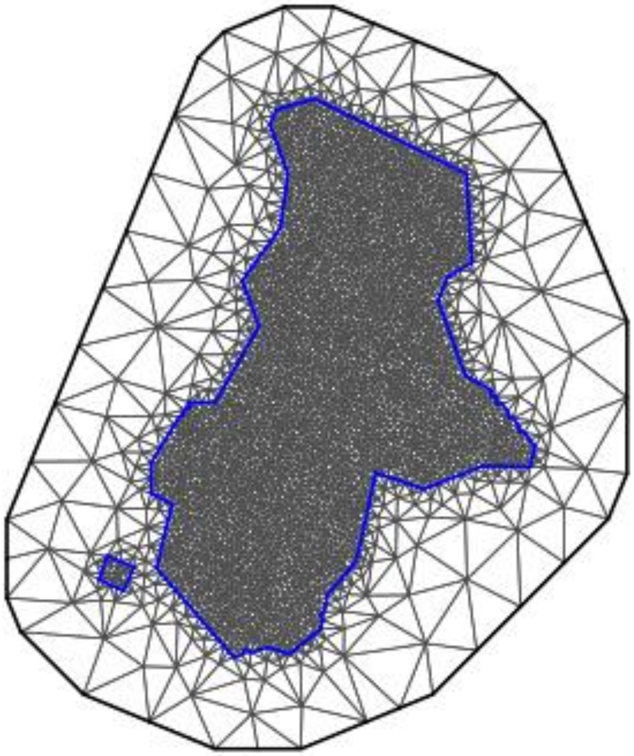

Figure S 7. Posterior means and 95% uncertainty intervals

Pixels with fewer than ten people per 1-km² and classified as "barren or sparsely vegetated" are colored in grey.

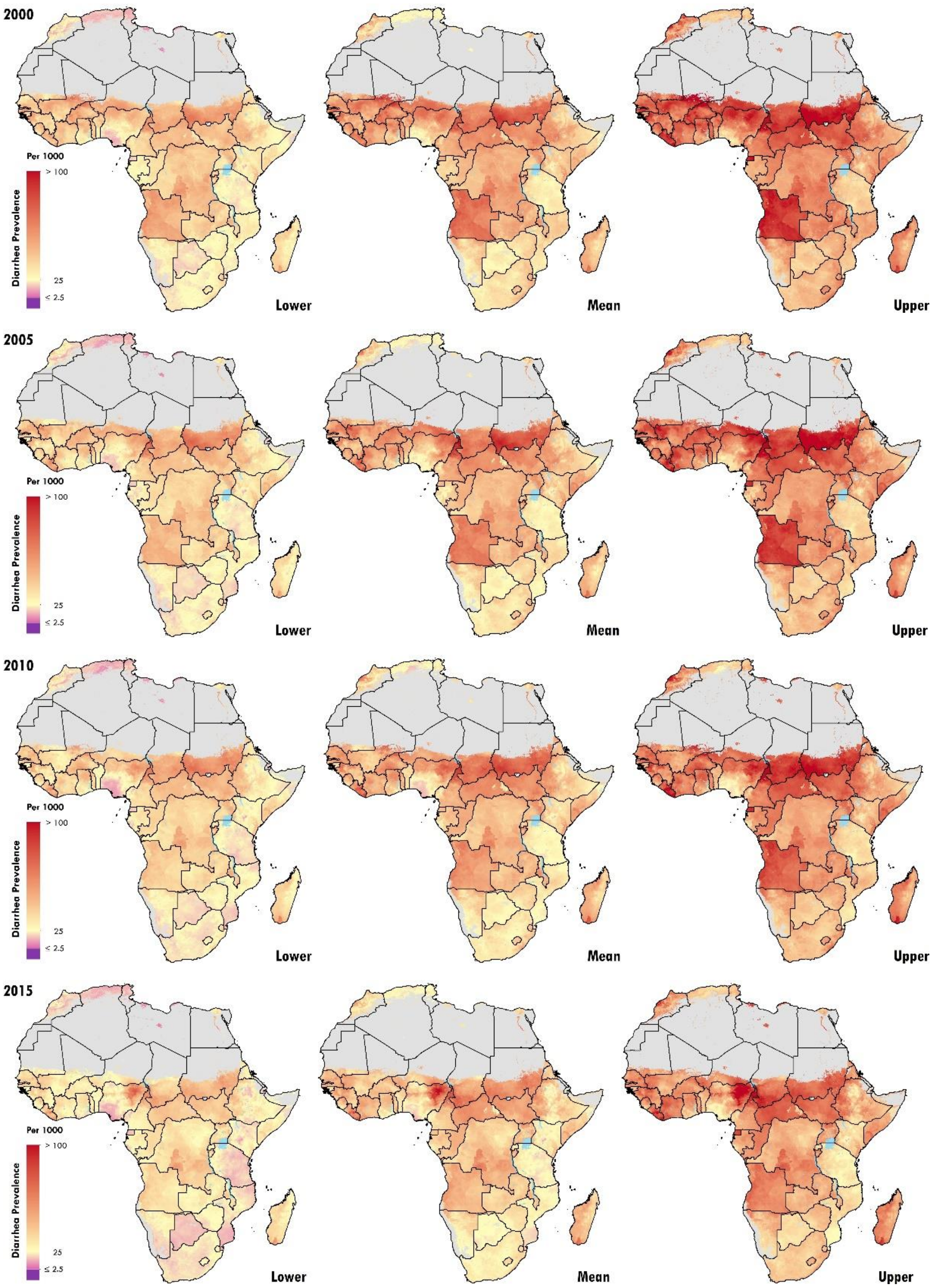

Figure S 8. Plots of Diarrhea prevalence absolute error in Africa

Color indicates magnitude of in-sample error, size of the points represents the sample size of the observed survey cluster or pseudo-cluster, and transparency represents the weight of the survey cluster or pseudo-cluster.

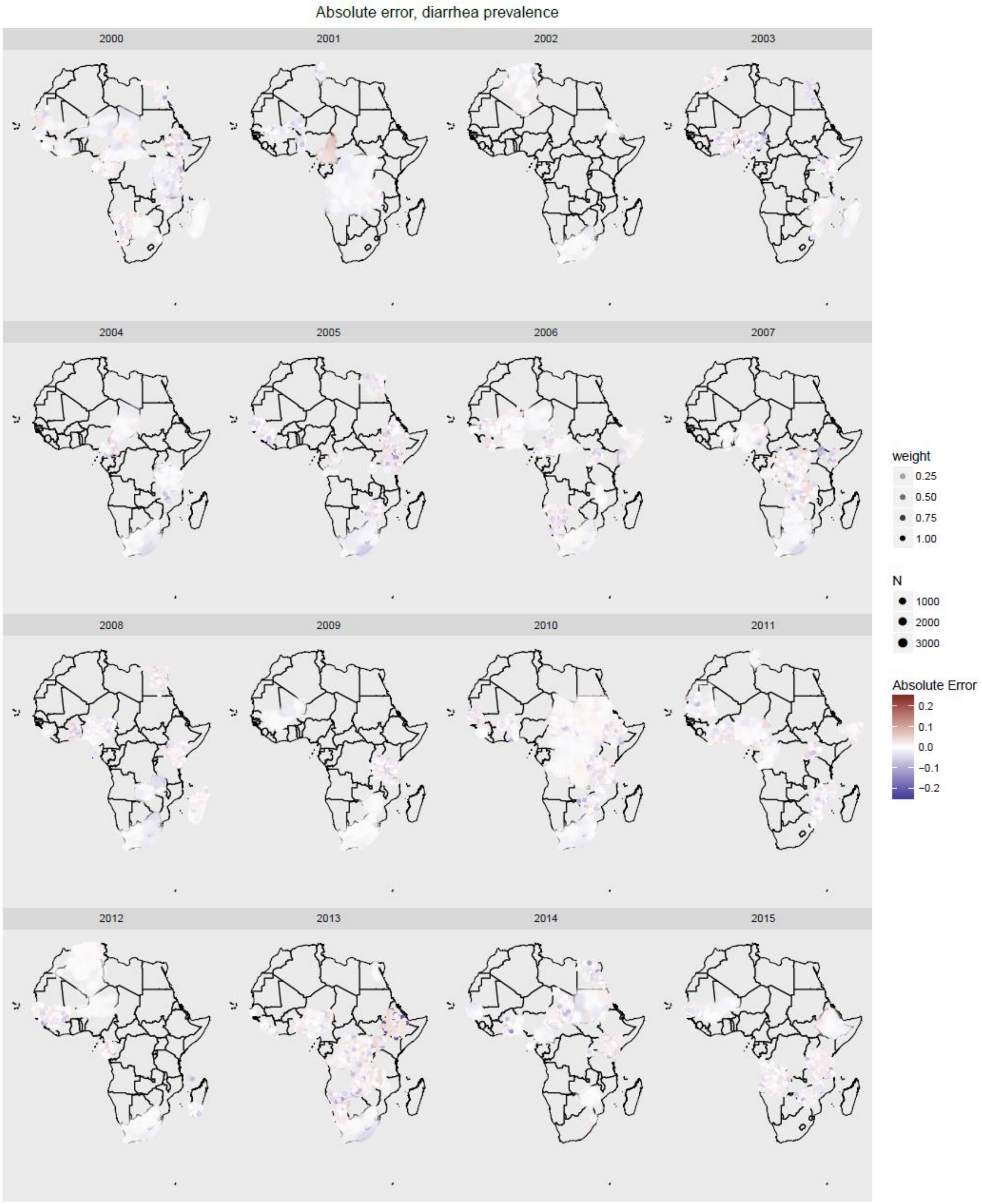

Figure S 9. Admin 0 aggregation

Comparison of out-of-sample predictions aggregated to admin 0 plotted against admin 0 aggregated data observations using quadtree (a) and second administrative unit (b) spatial holdout strategies. SS denotes the sample size of the aggregated data.

(a)

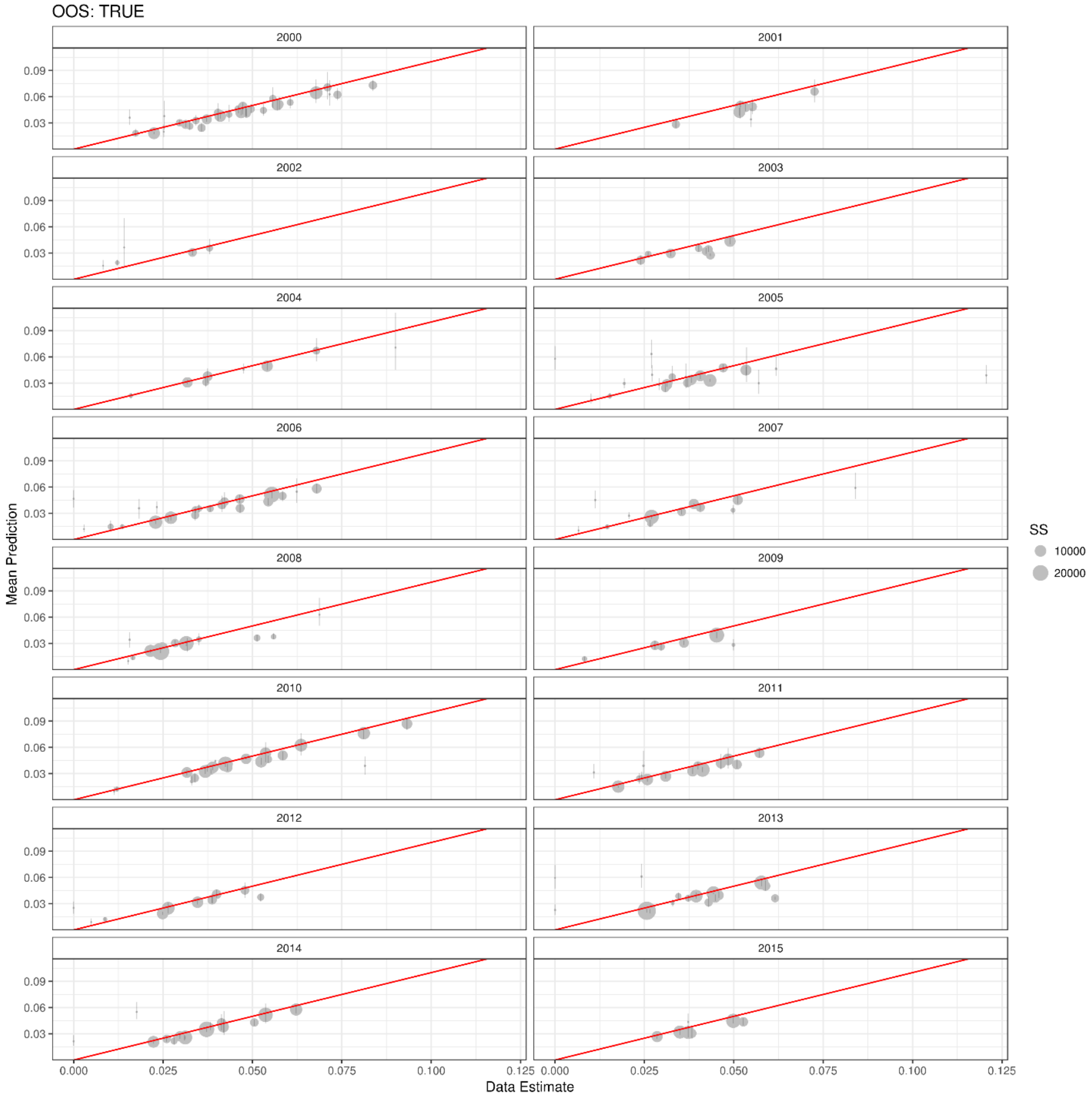

(b)

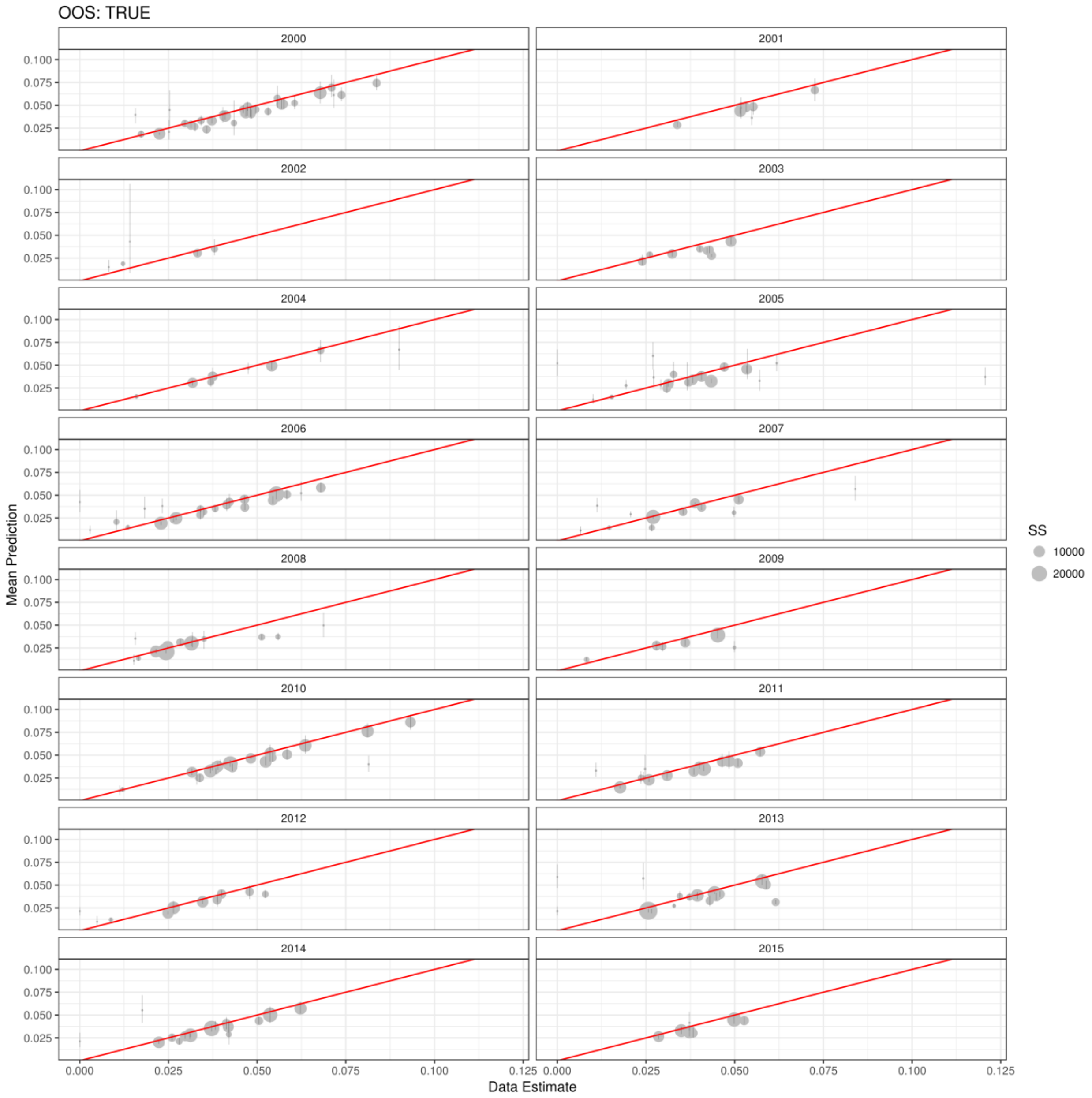
OOS: TRUE
2000
2001
2002
2003
2004
2005
2006
2007
2008
2009
2010
2011
2012
2013
2014
2015
Mean Prediction
0.100
0.075
0.050
0.025
Data Estimate
0.000
0.025
0.050
0.075
0.100
0.125
SS
10000
20000

Figure S 10. First administrative subdivision aggregation

Comparison of out-of-sample predictions aggregated to admin 1 plotted against admin 1 aggregated data observations using quadtree (a) and second administrative unit (b) spatial holdout strategies. SS denotes the sample size of the aggregated data.

(a)

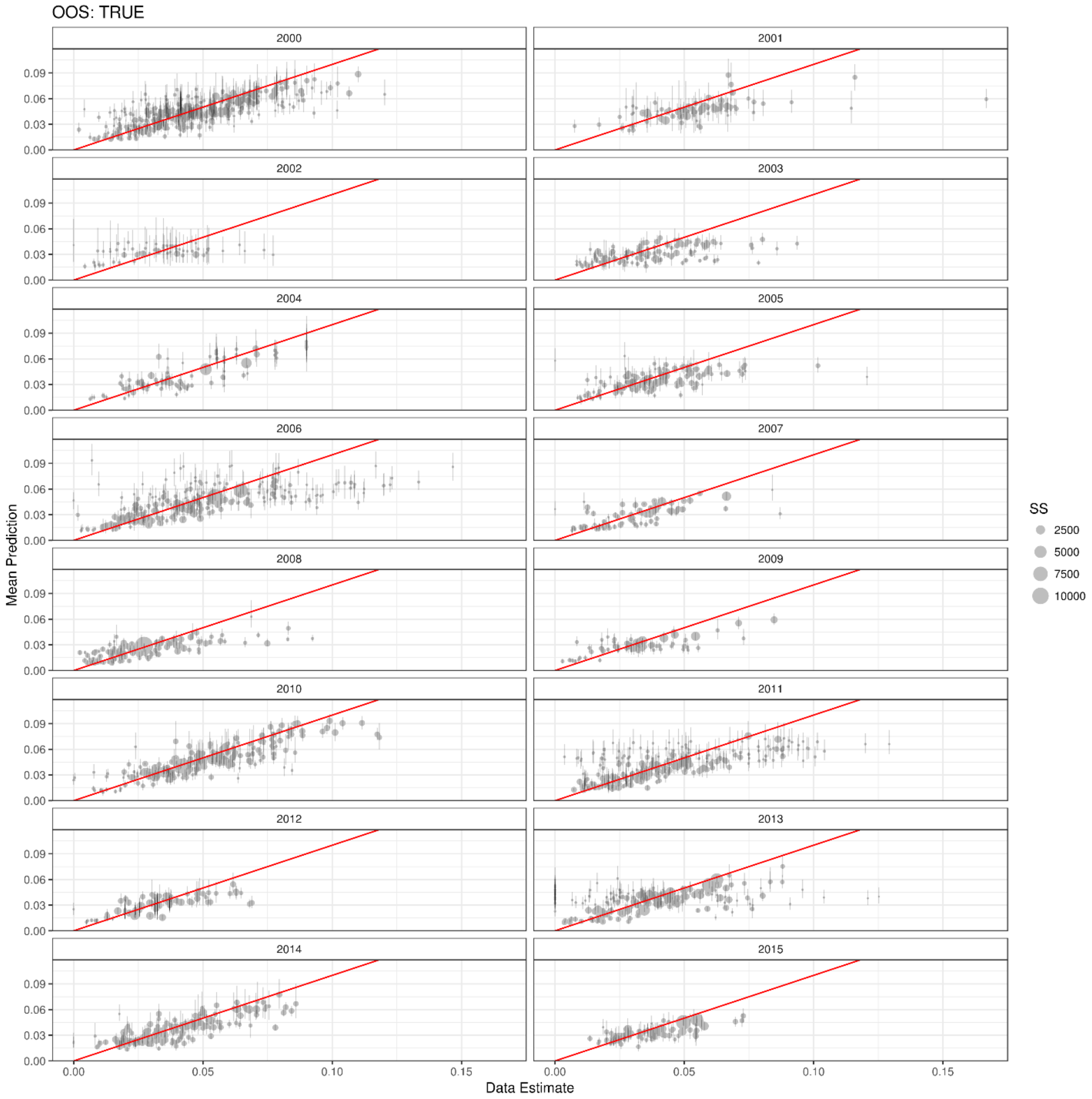

452 (b)

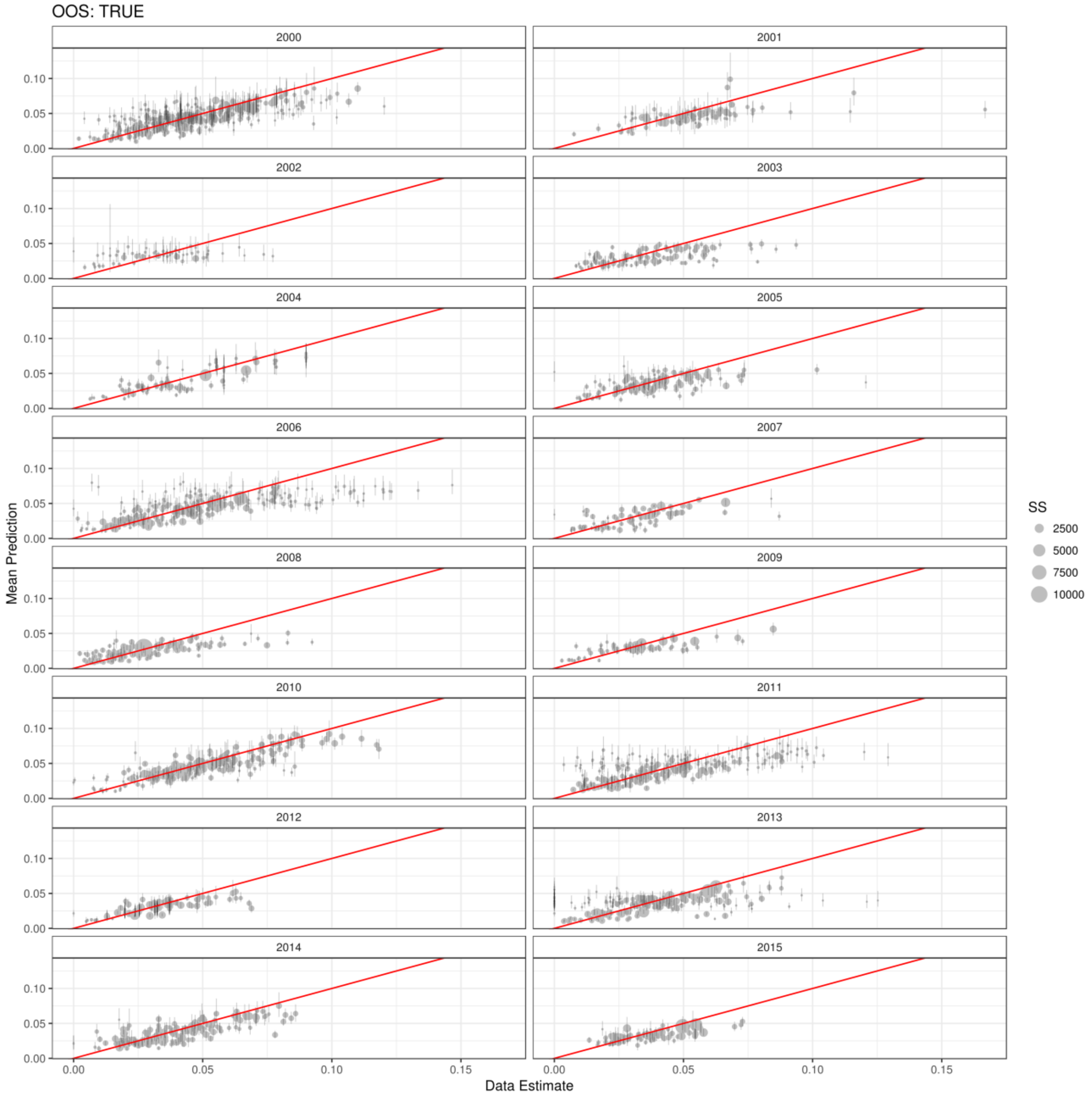

OOS: TRUE
2000
2001
2002
2003
2004
2005
2006
2007
2008
2009
2010
2011
2012
2013
2014
2015
0.00
0.05
0.10
0.15
Mean Prediction
Data Estimate
SS
2500
5000
7500
10000

453

Figure S 11. Second administrative subdivision aggregation

Comparison of out-of-sample predictions aggregated to admin 2 plotted against admin 2 aggregated data observations using quadtree (a) and second administrative unit (b) spatial holdout strategies. SS denotes the sample size of the aggregated data.

(a)

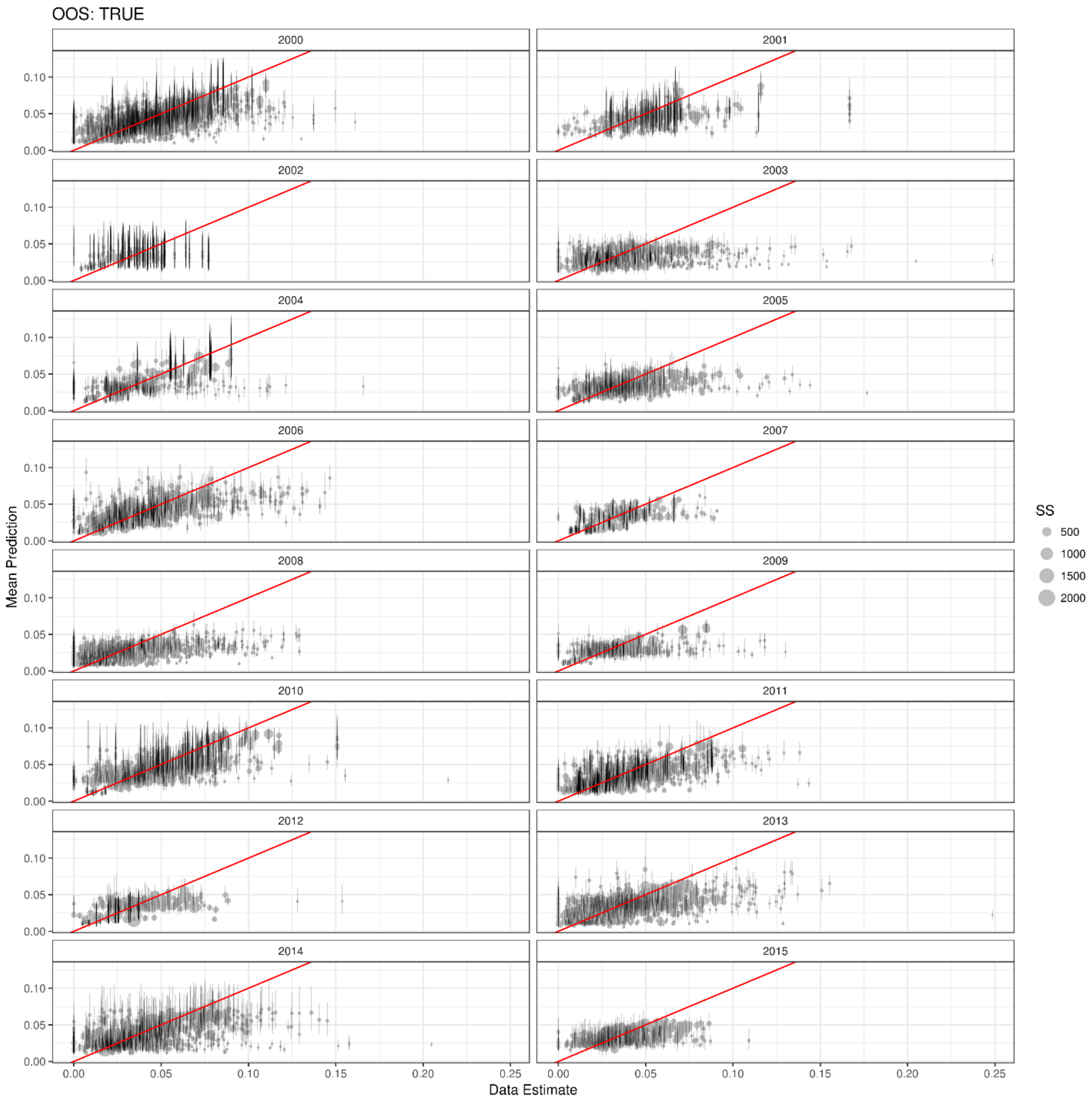

(b)

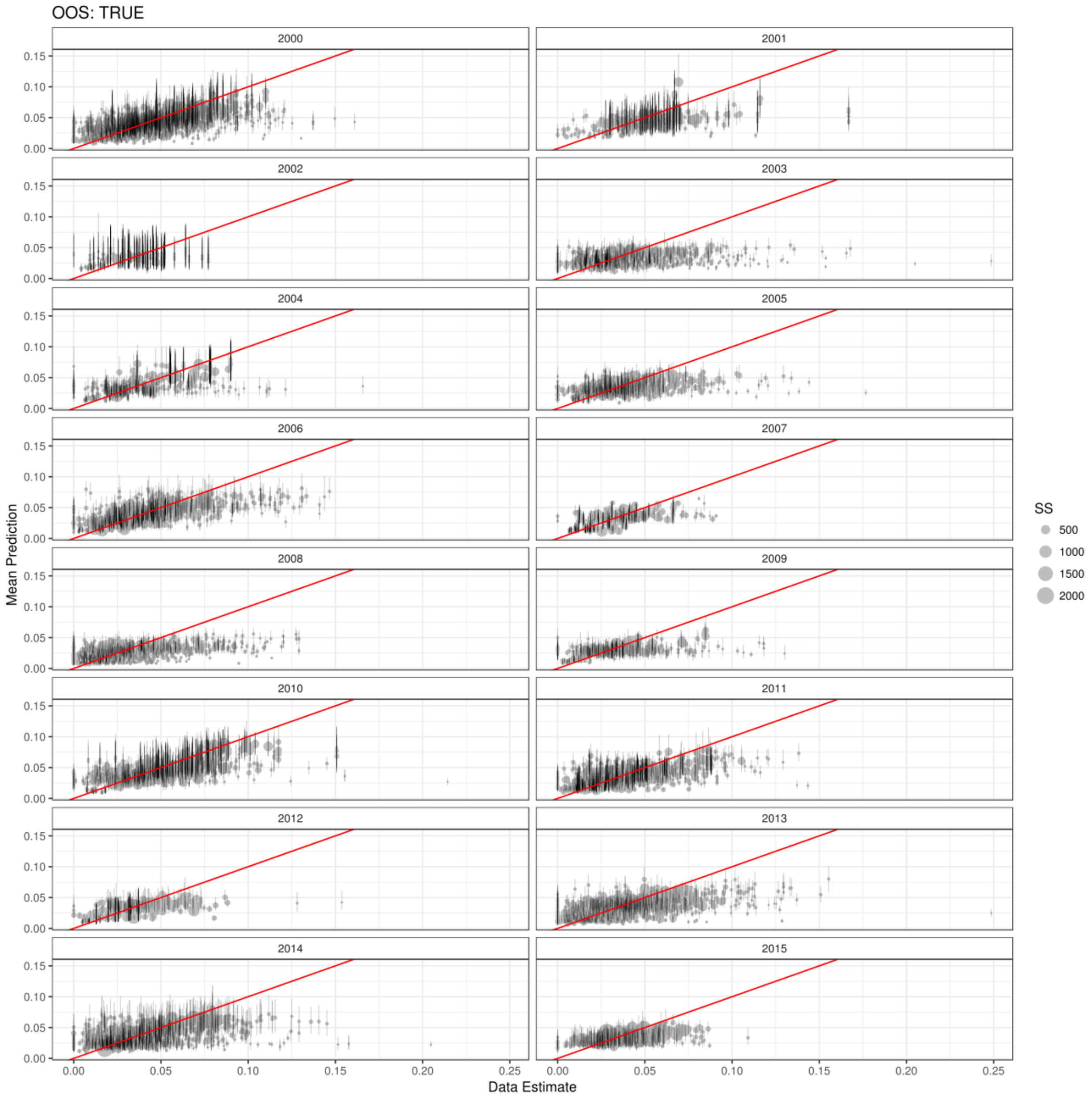
OOS: TRUE
2000
2001
2002
2003
2004
2005
2006
2007
2008
2009
2010
2011
2012
2013
2014
2015
Mean Prediction
0.00
0.05
0.10
0.15
Data Estimate
0.00
0.05
0.10
0.15
0.20
0.25
SS
500
1000
1500
2000

Figure S 12. Comparison of aggregated MBG estimates to GBD 2016 diarrhea prevalence estimates

Note that our models and GBD 2016 datasets overlap but are not identical

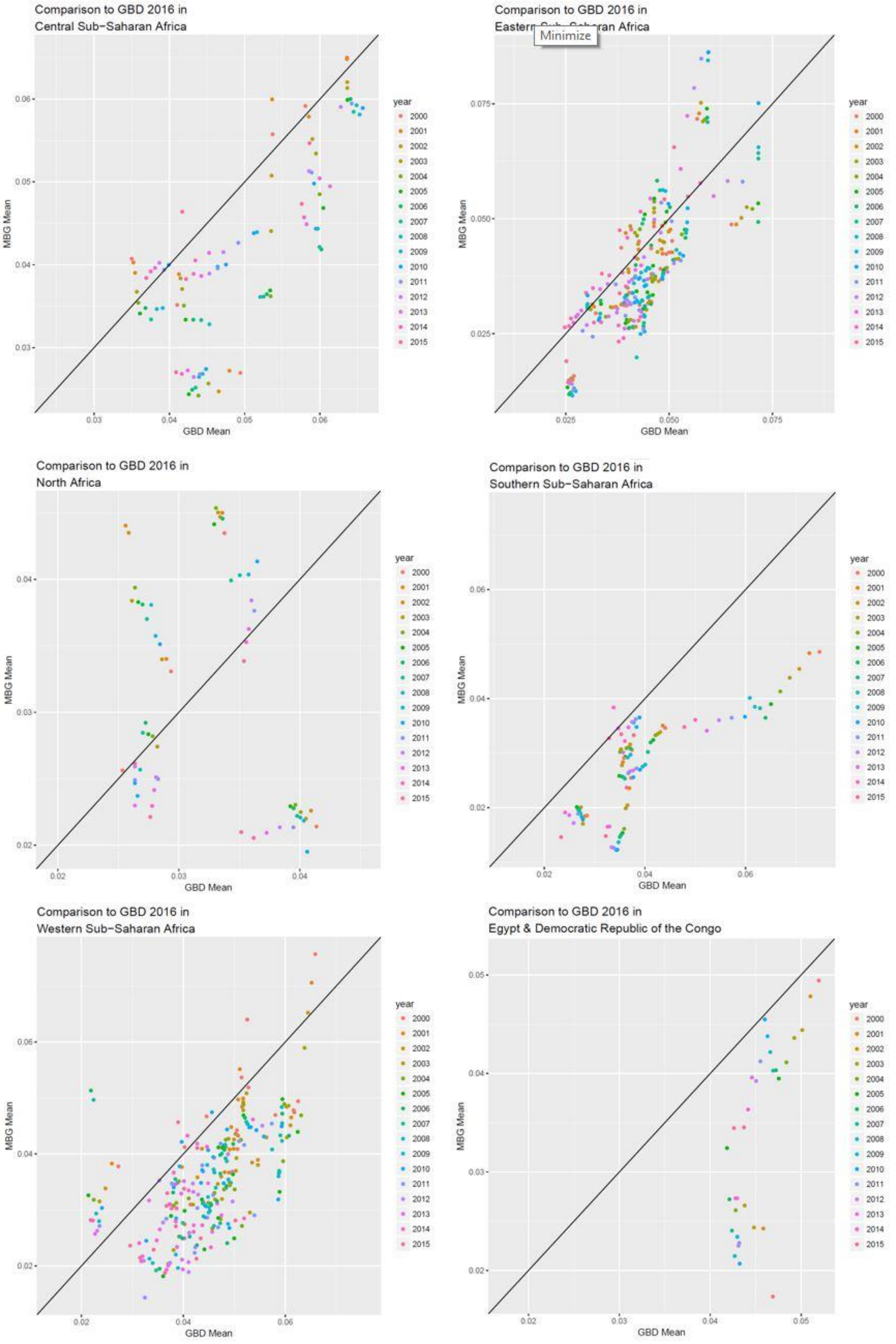

Figure S 13. First administrative subdivision Comparison of MBG estimates to DHS estimates

95% uncertainty intervals are plotted along with the aggregated MBG estimates. "Data estimate" refers to the survey weighted estimate of diarrhea prevalence from the DHS microdata. Note that our model includes more data than just the DHS surveys.

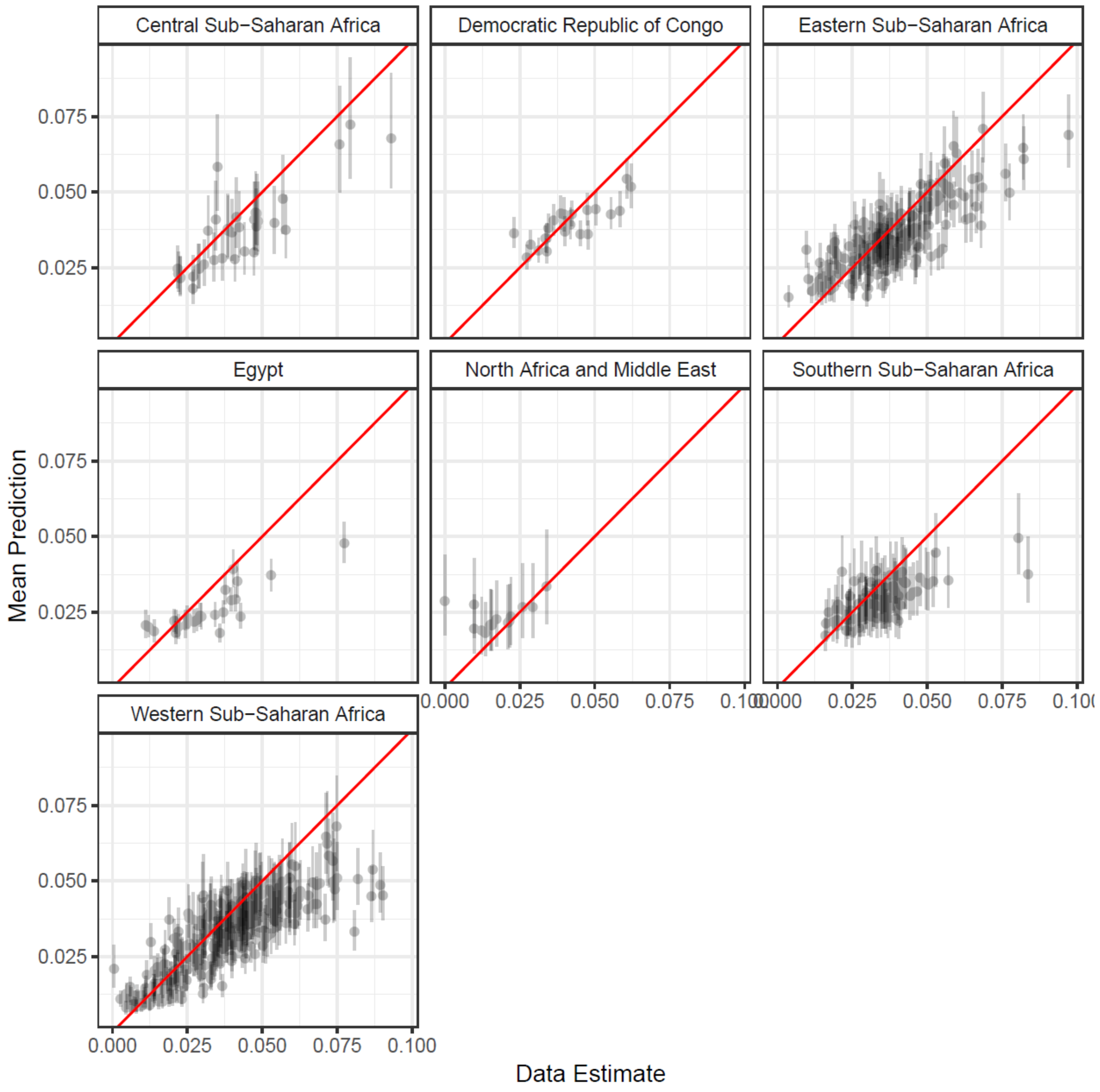

Figure S 14. Out of Sample Statistics of different model specifications

Coverage vs. RMSE, Bias vs. RMSE and Coverage vs. Bias are provided for five different model specifications under two spatial holdout schemes. Stacked + GP denotes a model (the model described in the methods section) where stacked generalization (stacked/ing) and Gaussian Process (GP) are used. Covs describes a model without stacking (e.g. covariates are left in their raw state) or the GP while the Stacked covs model uses stacking, but no GP. GP denotes a model with no covariates, but with the GP turned off while Raw + GP includes raw covariates and GP. In general, the figures show Gaussian Process and Stacking both improve out of sample validity relative to other formulations.

(a) Quad-tree

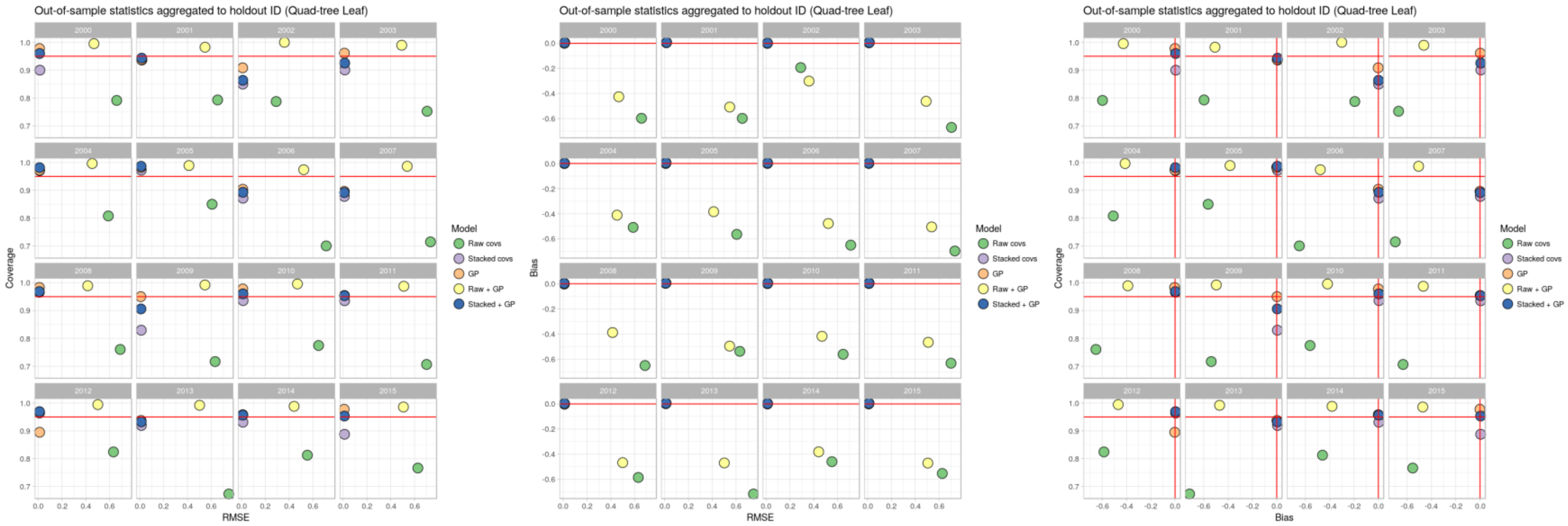

(b) Second Administrative Unit Holdout Scheme

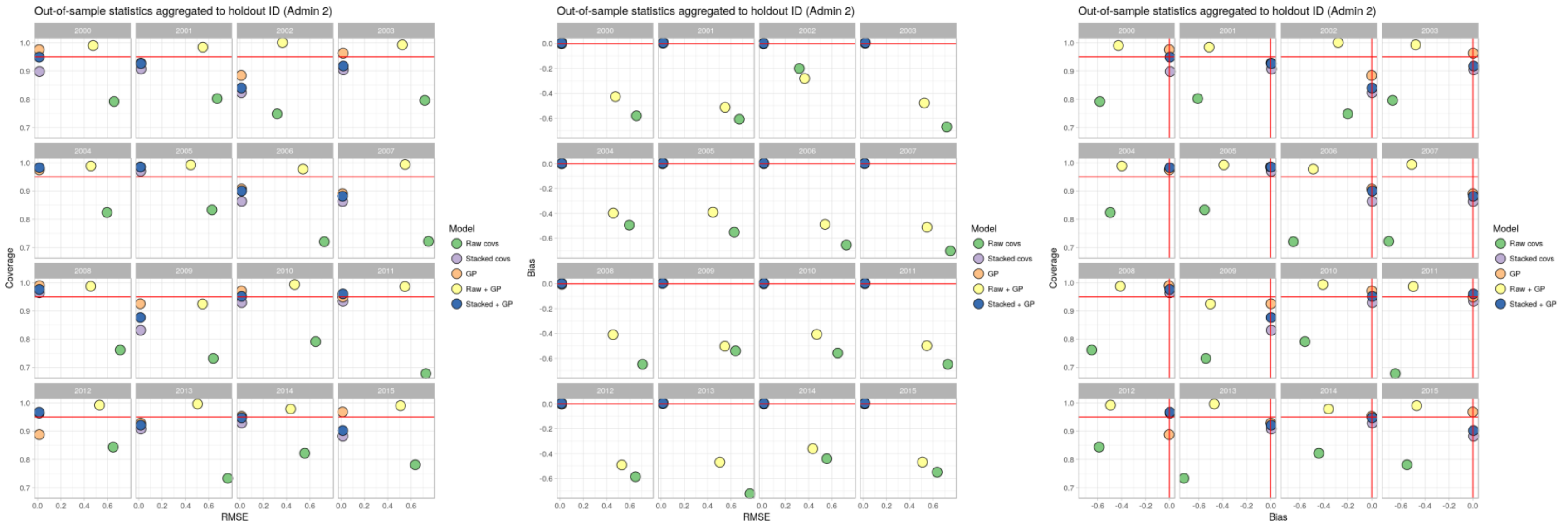

Figure S 15. Geospatial Modeling Flowchart

The geospatial modelling process consists of four sections. First (in blue), we compile all available survey data that can be referenced to a coordinate/points (e.g. survey cluster) or small polygon unit and calculate the diarrhea prevalence at the respective level. Data are then adjusted for seasonality and differential recall periods. Data matched to polygons are resampled into probable points using a k-means clustering algorithm. Covariates are subsequently merged to the points and pseudo points via a spatial join. Second (green), we use the point data and their associated covariates and a stacked generalization ensemble model. The children models, boosted regression trees, generalized additive models, and elastic net regression are fit using an internal 5-fold cross validation process. The cross validated predictions from each model then serve as the covariate values for the main/parent model (Spatio-temporal GPR model). The predictions from when the child models are fit on all the data (rather than 4/5ths implied by the cross validation) are then used to create posterior predictions of diarrhea prevalence in a 5-km^2 grid for the years 2000 – 2015. Third (purple and circled orange), we combine the predictions from step 2 and calibrate them such that the population weighted mean diarrhea prevalence for a particular country-year from our model matches the GBD estimates.[14,15] Finally (orange), we aggregate our estimates to first administrative units. Using these aggregate estimates and the previously calibrated pixel estimates, we are able to convert prevalence estimates into mortality and incidence estimates and otherwise generate maps of these values.

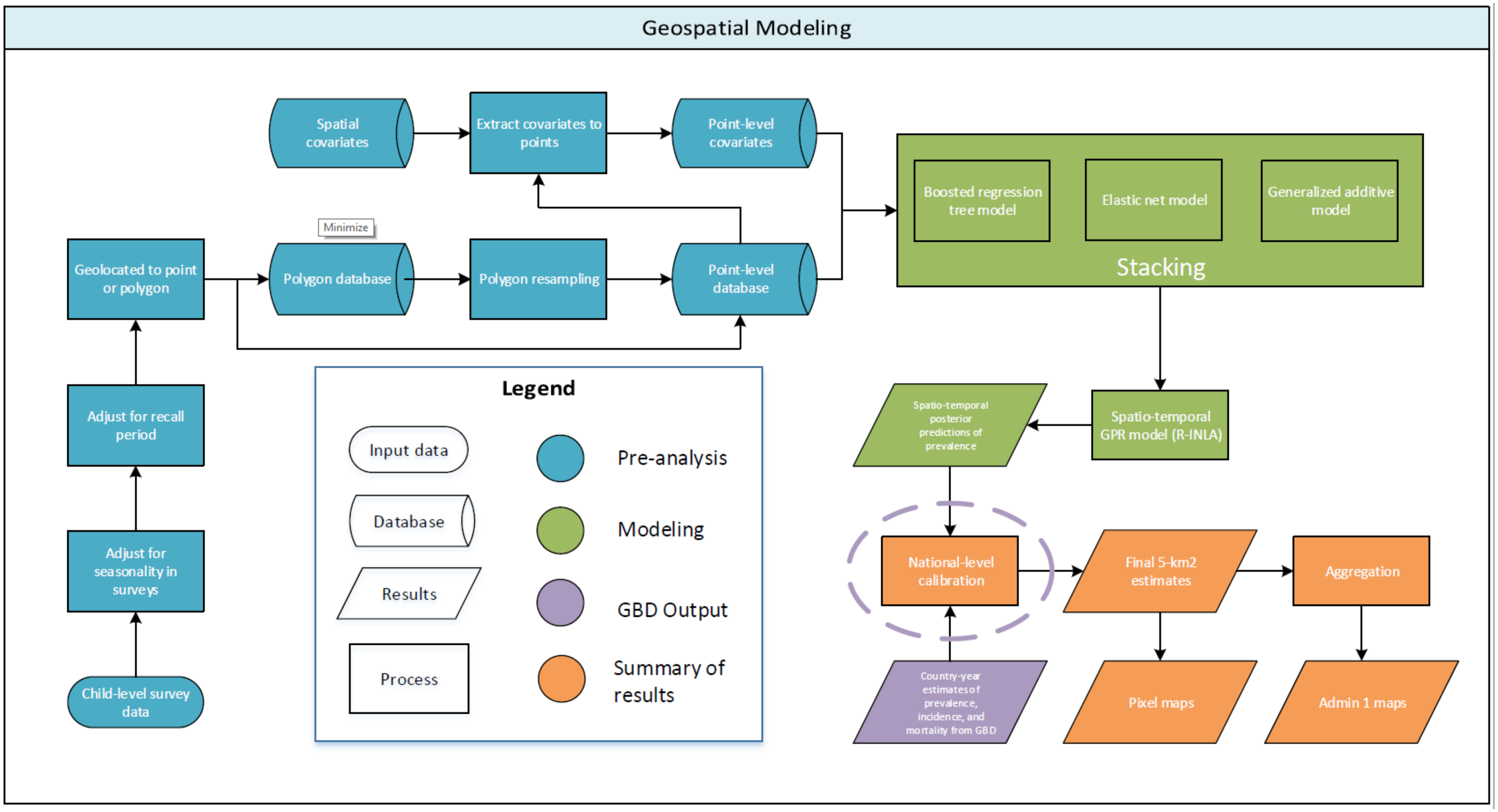

Geospatial Modeling
Spatial covariates
Extract covariates to points
Point-level covariates
Minimize
Geolocated to point or polygon
Polygon database
Polygon resampling
Point-level database
Boosted regression tree model
Elastic net model
Generalized additive model
Stacking
Adjust for recall period
Adjust for seasonality in surveys
Child-level survey data
Legend
Input data
Database
Results
Process
Pre-analysis
Modeling
GBD Output
Summary of results
Spatio-temporal posterior predictions of prevalence
Spatio-temporal GPR model (R-INLA)
National-level calibration
Final 5-km2 estimates
Aggregation
Country-year estimates of prevalence, incidence, and mortality from GBD
Pixel maps
Admin 1 maps

6.0 Supplementary Tables

Table S 1. Compliance for the Guidelines for Accurate and transparent Health Estimates Reporting 34
Table S 2. Household surveys and censuses used in mapping 36
Table S 3. Covariates used in mapping 42
Table S 4. Spatial hyperparameters priors by region. 47
Table S 5. Fitted parameters 48
Table S 6. Predictive metrics aggregated to admin 0 49
Table S 7. Predictive metrics aggregated to first administrative subdivision 49
Table S 8. Predictive metrics aggregated to Second administrative subdivision 49
Table S 9. Predictive metrics aggregated to holdout units 50

Table S 1. Compliance for the Guidelines for Accurate and transparent Health Estimates Reporting

Item #	Checklist item	Reported on page #
**Objectives and funding**		
**1**	Define the indicator(s), populations (including age, sex, and geographic entities), and time period(s) for which estimates were made.	6
**2**	List the funding sources for the work.	16
**Data Inputs**		
For all data inputs from multiple sources that are synthesized as part of the study:		
**3**	Describe how the data were identified and how the data were accessed.	6-7
**4**	Specify the inclusion and exclusion criteria. Identify all ad-hoc exclusions.	6;<br>S.4
**5**	Provide information on all included data sources and their main characteristics. For each data source used, report reference information or contact name/institution, population represented, data collection method, year(s) of data collection, sex and age range, diagnostic criteria or measurement method, and sample size, as relevant.	S.36-S.41
**6**	Identify and describe any categories of input data that have potentially important biases (e.g., based on characteristics listed in item 5).	6-8
For data inputs that contribute to the analysis but were not synthesized as part of the study:		
**7**	Describe and give sources for any other data inputs.	S.15;<br>S.42-S.46
For all data inputs:		
**8**	Provide all data inputs in a file format from which data can be efficiently extracted (e.g., a spreadsheet rather than a PDF), including all relevant meta-data listed in item 5. For any data inputs that cannot be shared because of ethical or legal reasons, such as third-party ownership, provide a contact name or the name of the institution that retains the right to the data.	8;<br>Available at ghdx.healthdata.org
**Data analysis**		
**9**	Provide a conceptual overview of the data analysis method. A diagram may be helpful.	6-8;<br>S.31-S.32
**10**	Provide a detailed description of all steps of the analysis, including mathematical formulae. This description should cover, as relevant, data cleaning, data pre-processing, data adjustments and weighting of data sources, and mathematical or statistical model(s).	6-8;<br>S.5-S.9
**11**	Describe how candidate models were evaluated and how the final model(s) were selected.	6-8;<br>S.10-S.11
**12**	Provide the results of an evaluation of model performance, if done, as well as the results of any relevant sensitivity analysis.	6-8;<br>S.10-S.11
**13**	Describe methods for calculating uncertainty of the estimates. State which sources of uncertainty were, and were not, accounted for in the	7-8<br>S.5-S.9

	uncertainty analysis.	
**14**	State how analytic or statistical source code used to generate estimates can be accessed.	8;<br>https://github.com
**Results and Discussion**		
**15**	Provide published estimates in a file format from which data can be efficiently extracted.	Raster files for spatial data and CSVs of admin 1 and admin 2 estimates to be made available at ghdx.healthdata.org
**16**	Report a quantitative measure of the uncertainty of the estimates (e.g. uncertainty intervals).	9-11;<br>S.19-S.20; S47-50
**17**	Interpret results in light of existing evidence. If updating a previous set of estimates, describe the reasons for changes in estimates.	12-15
**18**	Discuss limitations of the estimates. Include a discussion of any modelling assumptions or data limitations that affect interpretation of the estimates.	12-15

Table S 2. Household surveys and censuses used in mapping

Number identification (NID) can be used to locate a particular data source in the Global Health Data Exchange at http://ghdx.healthdata.org/.

Country	Survey start year	Source	Number Identification (NID)	Number of sampled children, 0-5	Number of geo-positioned survey clusters	Number of polygons (areal)
Algeria	2002	Arab League PAPFAM	627	2860	0	47
Algeria	2012	UNICEF MICS	210614	12545	0	7
Angola	2001	UNICEF MICS	687	5609	0	18
Angola	2015	DHS Program	218555	13463	625	0
Benin	2001	DHS Program	18950	4617	247	0
Benin	2006	DHS Program	18959	13731	0	12
Benin	2011	DHS Program	79839	12462	746	0
Benin	2014	UNICEF MICS	206075	10427	0	12
Botswana	2000	UNICEF MICS	1404	2651	0	14
Botswana	2007	National Survey	22125	2179	0	24
Burkina Faso	1998	DHS Program	19076	4910	208	0
Burkina Faso	2003	DHS Program	19088	9141	397	0
Burkina Faso	2006	UNICEF MICS	1927	5216	195	0
Burkina Faso	2010	DHS Program	19133	12820	541	0
Burundi	2005	UNICEF MICS	1981	5738	0	17
Burundi	2010	DHS Program	30431	7198	376	0
Cameroon	1998	DHS Program	19198	2010	0	10
Cameroon	2000	UNICEF MICS	2053	3191	0	10
Cameroon	2001	National Survey	2039	4794	0	11
Cameroon	2004	DHS Program	19211	6855	461	0
Cameroon	2006	UNICEF MICS	2063	5853	0	191
Cameroon	2011	DHS Program	19274	10317	577	0
Cameroon	2014	UNICEF MICS	244455	5877	0	12
Central African Republic	2000	UNICEF MICS	2209	12655	0	17
Central African Republic	2006	UNICEF MICS	2223	5091	0	12
Central African Republic	2010	UNICEF MICS	82832	9452	0	17
Chad	2000	UNICEF MICS	2244	4114	0	15

Chad	2004	DHS Program	19315	3627	0	9
Chad	2010	UNICEF MICS	76701	12358	0	60
Chad	2014	DHS Program	157025	16710	624	0
Comoros	2012	DHS Program	76850	2799	242	0
Congo	2005	DHS Program	19391	3303	0	12
Congo	2011	DHS Program	56151	7586	0	12
Cote d'Ivoire	1998	DHS Program	18531	1632	140	0
Cote d'Ivoire	2000	UNICEF MICS	26444	7879	0	11
Cote d'Ivoire	2011	DHS Program	18533	6680	341	0
Democratic Republic of the Congo	2001	UNICEF MICS	3161	9385	0	11
Democratic Republic of the Congo	2007	DHS Program	19381	7646	293	0
Democratic Republic of the Congo	2010	UNICEF MICS	26998	11036	360	10
Democratic Republic of the Congo	2013	DHS Program	76878	16225	491	0
Djibouti	2006	UNICEF MICS	3404	1902	89	0
Egypt	2000	DHS Program	19511	10901	987	0
Egypt	2003	DHS Program	19529	5486	880	0
Egypt	2005	DHS Program	19521	12723	1288	0
Egypt	2008	DHS Program	26842	10381	1221	0
Egypt	2013	UNICEF MICS	159617	4239	0	6
Egypt	2014	DHS Program	154897	15357	1738	0
Eritrea	2002	DHS Program	19539	5156	0	6
Ethiopia	1999	National Survey	133799	11149	0	11
Ethiopia	2000	DHS Program	19571	9256	533	0
Ethiopia	2005	DHS Program	19557	8794	528	0
Ethiopia	2010	DHS Program	21301	10324	571	0
Ethiopia	2013	World Bank LSMS ISA	235215	238	157	0
Ethiopia	2015	World Bank LSMS ISA	286657	200	137	0
Ethiopia	2016	DHS Program	218568	9667	622	0
Gabon	2000	DHS Program	19579	3747	0	40
Gabon	2012	DHS Program	76706	5375	330	0
Ghana	1998	DHS Program	19614	2960	397	0

Ghana	2003	DHS Program	19627	3432	407	0
Ghana	2006	UNICEF MICS	4694	3048	0	10
Ghana	2007	UNICEF MICS	160576	7270	0	4
Ghana	2008	DHS Program	21188	2711	401	0
Ghana	2010	UNICEF MICS	56241	396	81	0
Ghana	2011	UNICEF MICS	63993	6945	738	0
Ghana	2014	DHS Program	157027	5465	423	0
Guinea	1999	DHS Program	19670	4913	293	0
Guinea	2005	DHS Program	19683	5395	291	0
Guinea	2012	DHS Program	69761	6316	300	0
Guinea-Bissau	2000	UNICEF MICS	4808	5597	0	9
Guinea-Bissau	2006	UNICEF MICS	4818	5291	0	9
Guinea-Bissau	2014	UNICEF MICS	174049	7209	0	9
Kenya	1998	DHS Program	20132	3232	525	0
Kenya	2000	UNICEF MICS	7387	7152	815	0
Kenya	2003	DHS Program	20145	5275	398	0
Kenya	2005	National Survey	7375	8385	1315	0
Kenya	2007	UNICEF MICS	155335	953	78	0
Kenya	2008	DHS Program	21365	5594	397	0
Kenya	2008	UNICEF MICS	7401	11879	650	0
Kenya	2009	UNICEF MICS	56420	450	0	1
Kenya	2013	UNICEF MICS	203654	842	50	0
Kenya	2013	UNICEF MICS	203663	773	48	0
Kenya	2013	UNICEF MICS	203664	1066	57	0
Kenya	2014	DHS Program	157057	19778	1584	0
Lesotho	2004	DHS Program	20167	3031	379	0
Lesotho	2009	DHS Program	21382	3466	394	0
Lesotho	2014	DHS Program	157058	2824	396	0
Liberia	2006	DHS Program	20191	4920	291	0
Liberia	2013	DHS Program	77385	6806	322	0
Madagascar	2000	UNICEF MICS	27020	5747	0	6
Madagascar	2003	DHS Program	20223	2762	0	6
Madagascar	2008	DHS Program	21409	11349	585	0
Madagascar	2012	UNICEF MICS	125594	2993	127	0
Malawi	2000	DHS Program	20252	10185	559	0
Malawi	2004	DHS Program	20263	9713	520	0
Malawi	2006	UNICEF MICS	7919	20137	0	26
Malawi	2010	DHS Program	21393	17806	827	0

Malawi	2013	UNICEF MICS	161662	15960	0	31
Malawi	2015	DHS Program	218581	16246	850	0
Mali	2001	DHS Program	20315	10737	399	0
Mali	2006	DHS Program	20274	12145	405	0
Mali	2009	UNICEF MICS	270627	18008	0	9
Mali	2012	DHS Program	77388	9504	413	0
Mali	2015	UNICEF MICS	248224	13173	0	8
Mauritania	2000	DHS Program	20322	3263	0	13
Mauritania	2011	UNICEF MICS	152783	9121	0	194
Morocco	2003	DHS Program	20361	5600	480	0
Mozambique	2003	DHS Program	20394	6580	0	11
Mozambique	2011	DHS Program	55975	10146	609	0
Namibia	2000	DHS Program	20417	3388	257	0
Namibia	2006	DHS Program	20428	4353	486	0
Namibia	2013	DHS Program	150382	4449	536	0
Niger	1998	DHS Program	20537	4164	268	0
Niger	2000	UNICEF MICS	9439	4623	0	8
Niger	2006	DHS Program	20499	6932	0	8
Niger	2012	DHS Program	74393	9731	0	8
Nigeria	2003	DHS Program	20567	5032	359	0
Nigeria	2007	UNICEF MICS	9516	16487	0	37
Nigeria	2008	DHS Program	21433	25017	886	0
Nigeria	2011	UNICEF MICS	76703	16259	0	37
Nigeria	2013	DHS Program	77390	28095	889	0
Nigeria	2016	UNICEF MICS	218613	23408	0	37
Rwanda	2000	DHS Program	20722	6650	0	12
Rwanda	2000	UNICEF MICS	26930	3113	0	12
Rwanda	2005	DHS Program	20740	7551	456	0
Rwanda	2007	DHS Program	21222	4957	246	0
Rwanda	2010	DHS Program	56040	8418	492	0
Rwanda	2014	DHS Program	157063	7474	492	0
São Tomé & Príncipe	2000	UNICEF MICS	27055	2189	0	4
São Tomé & Príncipe	2008	DHS Program	26866	1653	0	7
São Tomé & Príncipe	2014	UNICEF MICS	214640	1852	0	7
Senegal	2005	DHS Program	26855	9416	366	0
Senegal	2010	DHS Program	56063	11137	385	0

Senegal	2012	DHS Program	111432	6319	200	0
Senegal	2014	DHS Program	191270	5034	0	14
Senegal	2015	DHS Program	218592	6360	214	0
Sierra Leone	2000	UNICEF MICS	11639	2669	0	4
Sierra Leone	2008	DHS Program	21258	4777	349	0
Sierra Leone	2010	UNICEF MICS	76700	6944	0	14
Sierra Leone	2013	DHS Program	131467	10254	435	0
Somalia	2006	UNICEF MICS	11774	6195	0	18
Somalia	2011	UNICEF MICS	91507	4658	259	0
Somalia	2011	UNICEF MICS	91508	4704	276	0
South Africa	1998	DHS Program	20796	4451	0	9
South Africa	2002	National Survey	115481	838	0	9
South Africa	2004	National Survey	11788	747	0	9
South Africa	2005	National Survey	11789	933	0	9
South Africa	2006	National Survey	115486	901	0	9
South Africa	2007	National Survey	11790	854	0	9
South Africa	2008	National Survey	115488	1071	0	9
South Africa	2009	National Survey	115489	1375	0	9
South Africa	2010	National Survey	115490	1055	0	9
South Africa	2012	National Survey	135534	750	0	9
South Sudan	2010	UNICEF MICS	32189	8197	0	10
Sudan	2000	UNICEF MICS	12243	20789	0	16
Sudan	2010	UNICEF MICS	153643	11952	0	15
Sudan	2014	UNICEF MICS	200617	11286	0	18
Swaziland	2000	UNICEF MICS	12320	3489	0	4
Swaziland	2006	DHS Program	20829	2359	268	0
Swaziland	2010	UNICEF MICS	30325	2535	0	4
Swaziland	2014	UNICEF MICS	200707	2210	0	4
United Republic of Tanzania	1999	DHS Program	20865	2646	173	0
United Republic of Tanzania	2000	National Survey	31740	987	0	20
United Republic of Tanzania	2004	DHS Program	20875	7302	0	26
United Republic of Tanzania	2004	World Bank CWIQ	31786	558	0	7
United Republic of Tanzania	2005	World Bank CWIQ	31797	178	0	2

United Republic of Tanzania	2009	DHS Program	21331	7053	458	0
United Republic of Tanzania	2010	World Bank LSMS ISA	81005	2499	0	125
United Republic of Tanzania	2015	DHS Program	218593	9445	608	0
The Gambia	2000	UNICEF MICS	3922	3624	0	8
The Gambia	2013	DHS Program	77384	6748	0	37
Togo	1998	DHS Program	20909	3838	283	0
Togo	2006	UNICEF MICS	12896	3499	0	6
Togo	2010	UNICEF MICS	40021	4060	0	6
Togo	2013	DHS Program	77515	6464	330	0
Tunisia	2001	Arab League PAPFAM	12978	1698	0	24
Tunisia	2011	UNICEF MICS	76709	2692	0	9
Uganda	2000	DHS Program	20993	5906	270	0
Uganda	2006	DHS Program	21014	6718	336	0
Uganda	2011	DHS Program	56021	7073	400	0
Uganda	2013	World Bank LSMS ISA	264959	2104	0	315
Zambia	1999	UNICEF MICS	14122	4453	0	71
Zambia	2001	DHS Program	21102	5861	0	72
Zambia	2007	DHS Program	21117	5720	319	0
Zambia	2008	Global Fund HH Survey	26702	1474	0	9
Zambia	2013	DHS Program	77516	12470	719	0
Zimbabwe	1999	DHS Program	21151	3076	219	0
Zimbabwe	2005	DHS Program	21163	4701	396	0
Zimbabwe	2009	UNICEF MICS	35493	5996	0	10
Zimbabwe	2010	DHS Program	55992	4880	393	0
Zimbabwe	2014	UNICEF MICS	152720	9025	0	10
Zimbabwe	2015	DHS Program	157066	5704	399	0

Table S 3. Covariates used in mapping

A variety of socioeconomic and environmental variables were used to predict diarrhea prevalence. Where available, the finest spatio-temporal resolution of gridded data sets was used.

Covariate	Temporal resolution	Source	Reference
Aridity	Annual	Climatic Research Unit Time-Series (CRUTS)	Harris, I., Jones, P. d., Osborn, T. j. & Lister, D. h. Updated high-resolution grids of monthly climatic observations – the CRU TS3.10 dataset. *Int. J. Climatol*. **34**, 623–642 (2014).<br><br>University of East Anglia. Climatic Research Unit TS v. 3.24 dataset. Available at: https://crudata.uea.ac.uk/cru/data/hrg/cru_ts_3.24.01/. (Accessed: 24th July 2017).
Average daily mean temperature	Annual	CRUTS	Harris, I., Jones, P. d., Osborn, T. j. & Lister, D. h. Updated high-resolution grids of monthly climatic observations – the CRU TS3.10 dataset. *Int. J. Climatol*. **34**, 623–642 (2014).<br><br>University of East Anglia. Climatic Research Unit TS v. 3.24 dataset. Available at: https://crudata.uea.ac.uk/cru/data/hrg/cru_ts_3.24.01/. (Accessed: 24th July 2017).
Average Land Surface Temperature (LST)	Annual	MODIS	USGS & NASA. Land surface temperature and emissivity 8-day L3 global 1km MOD11A2 dataset. Available at: https://lpdaac.usgs.gov/dataset_discovery/modis/modis_products_table/mod11a2. (Accessed: 24th July 2017)<br><br>Wan, Z. MODIS \Land-Surface Temperature Algorithm Theoretical Basis Document (LST ATBD).<br><br>Weiss, D. J. et al. An effective approach for gap-filling continental scale remotely sensed time-series. Isprs J. Photogramm. Remote Sens. 98, 106–118 (2014).
Cropland area	Static	Earth Stat	Ramankutty, N., Evan, A.T., Monfreda, C. & Foley, J.A. Farming the planet: 1. Geographic distribution of global agricultural lands in the year 2000. Global Biogeochemical Cycles 22, GB1003, doi:10.1029/2007GB002952 (2008).<br><br>Earth Stat. Download data. Available at http://www.earthstat.org/data-download/. (Accessed: 19th April 2017)

Dependency ratio (Dependents to working age adults)	Static	WorldPop (derived)	Lloyd, C. T., Sorichetta, A. & Tatem, A. J. High resolution global gridded data for use in population studies. Sci. Data 4, sdata20171 (2017).<br><br>World Pop. Get data. Available at: http://www.worldpop.org.uk/data/get_data/. (Accessed: 25th July 2017)
Distance to rivers and lakes	Static	Natural Earth Data (derived)	Natural Earth. Rivers and lake centerlines dataset. Available at: http://www.naturalearthdata.com/downloads/10m-physical-vectors/10m-rivers-lake-centerlines/. (Accessed: 24th July 2017)
Diurnal difference in LST	Annual	MODIS	USGS & NASA. Land surface temperature and emissivity 8-day L3 global 1km MOD11A2 dataset. Available at: https://lpdaac.usgs.gov/dataset_discovery/modis/modis_products_table/mod11a2. (Accessed: 24th July 2017)<br><br>Wan, Z. MODIS Land-Surface Temperature Algorithm Theoretical Basis Document (LST ATBD).<br><br>Weiss, D. J. et al. An effective approach for gap-filling continental scale remotely sensed time-series. Isprs J. Photogramm. Remote Sens. 98, 106–118 (2014).
Elevation	Static	NOAA	Hastings, David A., and Paula K. Dunbar. Global Land One-kilometer Base Elevation (GLOBE) Digital Elevation Model, Documentation, Volume 1.0. Key to Geophysical Records Documentation (KGRD) 34. National Oceanic and Atmospheric Administration, National Geophysical Data Center, 325 Broadway, Boulder, Colorado 80303, U.S.A (1999).<br><br>GLOBE Task Team and others (Hastings, David A., Paula K. Dunbar, Gerald M. Elphingstone, Mark Bootz, Hiroshi Murakami, Hiroshi Maruyama, Hiroshi Masaharu, Peter Holland, John Payne, Nevin A. Bryant, Thomas L. Logan, J.-P. Muller, Gunter Schreier, and John S. MacDonald), eds., 1999. The Global Land One-kilometer Base Elevation (GLOBE) Digital Elevation Model, Version 1.0. National Oceanic and Atmospheric Administration, National Geophysical Data Center, 325 Broadway, Boulder, Colorado 80303, U.S.A. Available at: https://www.ngdc.noaa.gov/mgg/topo/globe.html. (Accessed: 16th February 2017)

Enhanced Vegetation Index (EVI)	Annual	MODIS	Huete, A., Justice, C. & van Leeuwen, W. MODIS vegetation index (MOD 13) algorithm theoretical basis document. (1999).<br><br>USGS & NASA. Vegetation indices 16-Day L3 global 500m MOD13A1 dataset. Available at: https://lpdaac.usgs.gov/dataset_discovery/modis/modis_products_table/mod13a1. (Accessed: 25th July 2017)<br><br>Weiss, D. J. et al. An effective approach for gap-filling continental scale remotely sensed time-series. Isprs J. Photogramm. Remote Sens. 98, 106–118 (2014).
Fertility	Annual	WorldPop (derived)	Lloyd, C. T., Sorichetta, A. & Tatem, A. J. High resolution global gridded data for use in population studies. Sci. Data 4, sdata20171 (2017).<br><br>World Pop. Get data. Available at: http://www.worldpop.org.uk/data/get_data/. (Accessed: 25th July 2017)
Growing season length	Static	FAO	FAO. GAEZ - Global Agro-Ecological Zones data portal. Available at: http://www.fao.org/nr/gaez/about-data-portal/en/. (Accessed: 25th July 2017)<br><br>FAO. GAEZ - Global Agro-Ecological Zones users guide. (2012).
Irrigation	Static	University of Frankfurt	Goethe-Universität. Generation of a digital global map of irrigation areas. Available at: https://www.uni-frankfurt.de/45218039/Global_Irrigation_Map. (Accessed: 25th July 2017)
Lag-distributed income	Annual	Global Burden of Disease study	Global Burden of Disease Collaborative Network. Global Burden of Disease Study 2016 (GBD 2016) Covariates 1980-2016. Seattle, United States: Institute for Health Metrics and Evaluation (IHME), 2017.<br><br>Institute for Health Metrics and Evaluation. Global Health Data Exchange. Available at: http://internal-ghdx.healthdata.org/gbd-2016/data-input-sources.
Maternal education	Annual	Internally modelled	Currently in press, correspondence to Simon Hay.

Modified Functional Attribute Diversity (MFAD) of food commodities	Static	Herrero et al (modelled)	Herrero, M. et al. Farming and the geography of nutrient production for human use: a transdisciplinary analysis. Lancet Planet. Health 1, e33–e42 (2017).
Nighttime lights	Annual	NOAA DMSP	Savory et al. Intercalibration and Gaussian Process Modeling of Nighttime Lights Imagery for Measuring Urbanization Trends in Africa 2000– 2013. Remote Sens. 9, (2017).
Outdoor air pollution (PM2.5 concentration)	Annual	Global Burden of Disease study	Global Burden of Disease Collaborative Network. Global Burden of Disease Study 2016 (GBD 2016) Covariates 1980-2016. Seattle, United States: Institute for Health Metrics and Evaluation (IHME), 2017.<br><br>Institute for Health Metrics and Evaluation. Global Health Data Exchange. Available at: http://internal-ghdx.healthdata.org/gbd-2016/data-input-sources.
Population	Annual	WorldPop	Lloyd, C. T., Sorichetta, A. & Tatem, A. J. High resolution global gridded data for use in population studies. Sci. Data 4, sdata20171 (2017).<br><br>World Pop. Get data. Available at: http://www.worldpop.org.uk/data/get_data/. (Accessed: 25th July 2017)
Precipitation	Annual	CRUTS	Harris, I., Jones, P. d., Osborn, T. j. & Lister, D. h. Updated high-resolution grids of monthly climatic observations – the CRU TS3.10 dataset. *Int. J. Climatol*. **34**, 623–642 (2014).<br><br>University of East Anglia. Climatic Research Unit TS v. 3.24 dataset. Available at: https://crudata.uea.ac.uk/cru/data/hrg/cru_ts_3.24.01/. (Accessed: 24th July 2017).
Sanitation	Annual	Global Burden of Disease study	Global Burden of Disease Collaborative Network. Global Burden of Disease Study 2016 (GBD 2016) Covariates 1980-2016. Seattle, United States: Institute for Health Metrics and Evaluation (IHME), 2017.<br><br>Institute for Health Metrics and Evaluation. Global Health Data Exchange. Available at: http://internal-ghdx.healthdata.org/gbd-2016/data-input-sources.
Travel time to nearest settlement >50,000 inhabitants	Static	Malaria Atlas Project, Oxford Big Data Institute, Li Ka Shing Centre for Health	Currently in press, correspondence to Daniel J. Weiss.

		Information and Discovery, University of Oxford	
Urbanicity	Annual	European Commission/GHS	Pesaresi, M. et al. Operating procedure for the production of the Global Human Settlement Layer from Landsat data of the epochs 1975, 1990, 2000, and 2014. (Publications Office of the European Union, 2016).
Vaccine coverage - DPT3	Annual	Internally modelled	Currently unpublished, correspondence to Simon Hay.
Vaccine coverage - HIB3	Annual	Internally modelled	Currently unpublished, correspondence to Simon Hay.
Vaccine coverage - PCV3	Annual	Internally modelled	Currently unpublished, correspondence to Simon Hay.
Vaccine coverage - Rotavirus	Annual	Internally modelled	Currently unpublished, correspondence to Simon Hay.
Wet day frequency	Annual	CRUTS	Harris, I., Jones, P. d., Osborn, T. j. & Lister, D. h. Updated high-resolution grids of monthly climatic observations – the CRU TS3.10 dataset. *Int. J. Climatol*. **34**, 623–642 (2014).<br><br>University of East Anglia. Climatic Research Unit TS v. 3.24 dataset. Available at: https://crudata.uea.ac.uk/cru/data/hrg/cru_ts_3.24.01/. (Accessed: 24th July 2017).

Table S 4. Spatial hyperparameters priors by region.

Region	μ_{θ_1}	$\sigma^2_{\theta_1}$	μ_2	$\sigma^2_{\theta_2}$
**Central sub-Saharan Africa, adjusted**	-0.21743	10	-1.04808	10
**Democratic Republic of the Congo**	-0.48231	10	-0.7832	10
**Eastern sub-Saharan Africa, adjusted**	0.201328	10	-1.46684	10
**Egypt**	-0.73596	10	-0.52955	10
**Northern Africa, adjusted**	-0.00534	10	-1.26017	10
**Southern sub-Saharan Africa, adjusted**	0.07945	10	-1.34496	10
**Western sub-Saharan Africa, adjusted**	0.061271	10	-1.32678	10

Table S 5. Fitted parameters

Lower, median, and upper quantiles (0.025%, 0.50%, 0.975%) are displayed for the main parameters by region. The first four rows provide information on the fixed effects: the intercept (int) and the the covariates (gam, gbm, and enet) corresponding to the predicted ensemble rasters. Fitted values for the spatio-temporal field hyperparameters and the precisions (inverse variance) for our random effects are shown in the bottom four rows.

	Central sub-Saharan Africa quantiles			Demographic Republic of the Congo quantiles			Eastern sub-Saharan Africa quantiles			Egypt quantiles			Northern Africa quantiles			Southern sub-Saharan Africa quantiles			Western sub-Saharan Africa quantiles		
	**0.025**	**0.500**	**0.975**	**0.025**	**0.500**	**0.975**	**0.025**	**0.500**	**0.975**	**0.025**	**0.500**	**0.975**	**0.025**	**0.500**	**0.975**	**0.025**	**0.500**	**0.975**	**0.025**	**0.500**	**0.975**
**int**	-0.158	0.108	0.374	-0.004	0.098	0.200	-0.089	0.074	0.237	0.172	0.281	0.389	-0.373	0.079	0.524	-0.119	0.151	0.420	-0.020	0.147	0.315
**gam**	0.027	0.237	0.448	-0.677	-0.282	0.113	-0.012	0.158	0.328	-0.299	0.031	0.364	-0.242	0.462	1.158	-0.270	0.048	0.367	-0.301	-0.152	-0.003
**gbm**	0.465	0.568	0.671	0.540	0.696	0.851	0.504	0.584	0.663	0.409	0.538	0.667	0.485	0.806	1.128	0.458	0.575	0.693	0.635	0.708	0.782
**enet**	0.015	0.195	0.374	0.241	0.586	0.931	0.102	0.258	0.415	0.121	0.431	0.737	-0.926	-0.268	0.396	0.073	0.376	0.678	0.301	0.444	0.586
**Nominal Range**	0.297	2.094	3.276	1.508	2.460	3.905	1.034	1.437	1.896	-0.748	0.483	1.814	-0.671	1.951	4.528	1.662	3.011	4.125	0.374	0.646	0.916
**Nominal Variance**	-1.993	-0.985	0.327	-2.420	-1.468	-0.545	-1.632	-0.951	-0.379	-1.161	0.030	1.154	-2.211	1.116	5.853	-2.943	-1.762	0.168	-0.888	-0.570	-0.251
**Ar1 ϱ**	-0.995	-0.849	-0.091	-0.709	0.111	0.846	0.570	0.822	0.950	-0.977	-0.786	-0.046	-0.984	-0.051	0.983	-0.858	0.156	0.965	-0.671	-0.448	-0.146
**Precision for CTRY.ID**	2.464	5.361	11.472	N/A	N/A	N/A	4.728	8.723	15.106	N/A	N/A	N/A	1.308	3.446	8.430	2.578	5.601	11.330	4.606	8.796	15.725

Out of Sample Validation metrics, Tables S6-S9.

Table S 6. Predictive metrics aggregated to admin 0

Holdout Strategy	Year	Median SS	Mean Err.	RMSE	Corr.	95% Cov.
**Quadtree**	2000	3930	0.00414	0.00533	0.97479	0.95828
**Quadtree**	2005	178	0.00489	0.00657	0.83153	0.98352
**Quadtree**	2010	7198	0.00381	0.00486	0.98451	0.96171
**Quadtree**	2015	9100	0.00488	0.00534	0.97176	0.9554
**Admin 2**	2000	3931	0.00455	0.00571	0.97333	0.95454
**Admin 2**	2005	179	0.00465	0.00665	0.80355	0.98614
**Admin 2**	2010	7198	0.00415	0.0051	0.98534	0.94952
**Admin 2**	2015	9100	0.00468	0.0052	0.96588	0.89975

Table S 7. Predictive metrics aggregated to first administrative subdivision

Holdout Strategy	Year	Median SS	Mean Err.	RMSE	Corr.	95% Cov.
**Quadtree**	2000	215	0.00414	0.01039	0.86529	0.96471
**Quadtree**	2005	408	0.00489	0.01155	0.66283	0.98118
**Quadtree**	2010	580	0.00381	0.01022	0.8842	0.9577
**Quadtree**	2015	474	0.00488	0.00962	0.78969	0.95452
**Admin 2**	2000	215	0.00455	0.01089	0.85346	0.954
**Admin 2**	2005	409	0.00465	0.01176	0.63456	0.98236
**Admin 2**	2010	581	0.00415	0.01073	0.87328	0.9516
**Admin 2**	2015	474	0.00468	0.01008	0.73899	0.92923

Table S 8. Predictive metrics aggregated to Second administrative subdivision

Holdout Strategy	Year	Median SS	Mean Err.	RMSE	Corr.	95% Cov.
**Quadtree**	2000	35	0.00414	0.014	0.77362	0.96433
**Quadtree**	2005	58	0.00489	0.01678	0.49839	0.98517
**Quadtree**	2010	59	0.00381	0.01426	0.79465	0.95853
**Quadtree**	2015	65	0.00488	0.01389	0.62164	0.95079
**Admin 2**	2000	35	0.00455	0.01453	0.75586	0.95293
**Admin 2**	2005	58	0.00465	0.01692	0.48012	0.98253
**Admin 2**	2010	59	0.00415	0.01496	0.7722	0.94977
**Admin 2**	2015	65	0.00468	0.01443	0.5608	0.92981

Table S 9. Predictive metrics aggregated to holdout units

Holdout Strategy	Year	Median SS	Mean Err.	RMSE	Corr.	95% Cov.
**Quadtree**	2000	8	0.0042	0.01148	0.81014	0.95963
**Quadtree**	2005	6	0.00489	0.01032	0.61123	0.98658
**Quadtree**	2010	4	0.00381	0.01214	0.83243	0.96027
**Quadtree**	2015	11	0.00489	0.01034	0.68429	0.95298
**Admin 2**	2000	34	0.0046	0.01391	0.77206	0.94861
**Admin 2**	2005	44	0.00464	0.0151	0.51927	0.98459
**Admin 2**	2010	45	0.00415	0.01496	0.77211	0.95201
**Admin 2**	2015	75	0.00469	0.01436	0.56286	0.90159

7.0 Supplementary Appendix References

1. Lumley T. Analysis of complex survey samples. Journal of Statistical Software 2004;9(1):1–19.

2. Golding N, Burstein R, Longbottom J, et al. Mapping under-5 and neonatal mortality in Africa, 2000–15: a baseline analysis for the Sustainable Development Goals. The Lancet [Internet] 2017;Available from: http://www.sciencedirect.com/science/article/pii/S0140673617317580

3. Tatem AJ. WorldPop, open data for spatial demography. Sci Data 2017;4:sdata20174.

4. Osgood-Zimmerman A, Millear AI, Stubbs RW, et al. Mapping child growth failure in Africa between 2000 and 2015. Nature 2018;555(7694):41–7.

5. Bhatt S, Cameron E, Flaxman SR, Weiss DJ, Smith DL, Gething PW. Improved prediction accuracy for disease risk mapping using Gaussian process stacked generalization. J R Soc Interface 2017;14(134):20170520.

6. GBD 2015 Disease and Injury Incidence and Prevalence Collaborators, Arora M, Barber RM, et al. Global, regional, and national disability-adjusted life-years (DALYs) for 315 diseases and injuries and healthy life expectancy (HALE), 1990–2015: a systematic analysis for the Global Burden of Disease Study 2015. The Lancet 2016;388(10053):1603–58.

7. Stein ML. Interpolation of Spatial Data: Some Theory for Kriging [Internet]. Springer New York; 1999 [cited 2017 Oct 17]. Available from: //www.springer.com/us/book/9780387986296

8. Gelfand AE, Diggle P, Guttorp P, Fuentes M, editors. Handbook of Spatial Statistics. 1 edition. Boca Raton: CRC Press; 2010.

9. Rue H, Martino S, Chopin N. Approximate Bayesian inference for latent Gaussian models by using integrated nested Laplace approximations. J R Stat Soc Ser B Stat Methodol 2009;71(2):319–92.

10. Martins T, Simpson D, Lundgren F, Rue H. Bayesian computing with INLA: new features. Comput Stat Data Anal 2013;67:68–83.

11. Lidngren F, Rue H, Lindström J. An explicit link between Gaussian fields and Gaussian Markov random fields: the stochastic partial differential equation approach. J R Stat Soc Ser B Stat Methodol 2011;73(4):423–98.

12. Roberts DR, Bahn V, Ciuti S, Boyce MS, Elith J, Guillera-Arroita G. Cross-validation strategies for data with temporal, spatial, hierarchical, or phylogenetic structure. Ecography 2017;40(8):913–29.

13. The Global Administrative Unit layers (GAUL): Technical Aspects [Internet]. FAO. Available from: http://www.fao.org/geonetwork/srv/en/main.home

14. GBD 2016 Causes of Death Collaborators. Global, regional, and national age-sex specific mortality for 264 causes of death, 1980-2016: a systematic analysis for the Global Burden of Disease Study 2016. Lancet Lond Engl 2017;390(10100):1151–210.

15. GBD 2016 Disease and Injury Incidence and Prevalence Collaborators. Global, regional, and national incidence, prevalence, and years lived with disability for 328 diseases and injuries for 195 countries, 1990-2016: a systematic analysis for the Global Burden of Disease Study 2016. Lancet Lond Engl 2017;390(10100):1211–59.
